# Identifying Contextual Factors That Shape Cybersecurity Risk Perception for Assisted Living and Health Care Technologies and Wearables: Mixed Methods Study

**DOI:** 10.2196/64388

**Published:** 2025-03-19

**Authors:** Alvhild Skjelvik, Nicholas West, Matthias Görges

**Affiliations:** 1 Department of Information Security and Communication Technology Norwegian University of Science and Technology (NTNU) Gjøvik Norway; 2 Research Institute BC Children's Hospital Vancouver, BC Canada; 3 Department of Anesthesiology, Pharmacology and Therapeutics University of British Columbia Vancouver, BC Canada

**Keywords:** digital health, health care technology, cybersecurity, risk perception, health care stakeholders, contextual factors

## Abstract

**Background:**

Over the last decade, the health care technology landscape has expanded significantly, introducing new and innovative solutions to address health care needs. The implications of cybersecurity incidents in the health care context extend beyond data breaches to potentially harming individuals’ health and safety. Risk perception is influenced by various contextual factors, contributing to cybersecurity concerns that technological safeguards alone cannot address. Thus, it is imperative to study risk perceptions, contextual factors, and technological benefits to guide policy development, risk management, education, and implementation strategies.

**Objective:**

This study aims to investigate the differences in cybersecurity risk perception among various stakeholders in the health care sector in Norway and British Columbia (BC), Canada, and identify specific contextual factors that shape these perceptions. We expect to identify differences in risk perceptions for the explored health care technologies.

**Methods:**

We used a mixed methods approach comprising surveys and semistructured interviews to sample health care–related wearable technology stakeholders, including health care workers, patients (adults and adolescents) and their families, health authorities and hospital staff (biomedical engineers, information technology support, research staff), and device vendors/industry professionals in Norway and BC. Surveys explored information security scenarios based on the Behavioral-Cognitive Internet Security Questionnaire (BCISQ), risk perception, and contextualizing variables. We analyzed both survey data sets to summarize participants’ characteristics and responses to questions related to the BCISQ (behavior and attitude) and risk perception. Interviews were analyzed thematically using an inductive-deductive approach to explore risk perception and contextual factors.

**Results:**

Data from 274 survey respondents were available for analysis: 185 from Norway, including 139 (75.1%) females, and 89 from BC, including 57 (64%) females. A total of 45 respondents (31 in Norway and 14 in BC) participated in interviews. The BCISQ showed minor differences between locations; respondents demonstrated generally low-risk behavior and robust information security awareness. However, password simulation demonstrated discrepancies between self-assessed and “real” behavior by sharing or willingness to share passwords. Perceived risk is generally considered low, yet consequences of cybersecurity risks were evaluated as major but unlikely. Risk perception was stronger for assisted living and diabetes technologies than for smartwatches. The most important contextual factors shaping risk perceptions are human factors encompassing knowledge, competence, familiarity, feelings of dread, perceived benefit, and trust, as well as the technological factor of device functionality. Organizational and technological factors had lesser effects.

**Conclusions:**

We found minimal differences in behavior and risk perception among Norwegian and BC participants. Human factors and device functionality were most influential in shaping cybersecurity risk perceptions. Considering the rising need for assisted living technologies and wearables, insights into risk perceptions can strengthen risk management, awareness, and competence building. Further, it can address potential concerns among stakeholders to enable quicker technology adoption.

## Introduction

### Background

Over the last decade, the landscape of health care technology has expanded significantly, introducing innovative solutions to assist health care needs [1]. These advances have been instrumental in enhancing the efficiency of health care delivery, reducing operational costs, and optimizing timely management. The adoption of artificial intelligence–based solutions in the health care sector has accelerated through applications such as artificial intelligence–supported analysis of X-ray interpretation and blood sample evaluations [2-5]. Moreover, an emerging trend is using technology to facilitate remote patient care outside traditional health care environments such as hospitals [6,7]. This trend introduces multiple challenges, particularly in cybersecurity. Medical devices and assisted living technologies have been the subject of cybersecurity incidents [8-11]. Recently, several hundred insulin pumps were called back by the Food and Drug Administration (responsible for regulating medical devices in the United States) due to software weaknesses, resulting in the battery depletion of insulin pumps [12,13]. Although this was not an intended attack, it demonstrates the vulnerabilities in the technology that could be exploited for malicious purposes. Incidents resulting from intended attacks have also occurred, including attacks on home care services in Ontario, Canada, Norway, and Sweden, which jeopardized patient safety and their data [14,15].

A wide range of remote care technologies are available. These technologies collect and transfer data generated from patients, such as measurements and self-reported data, also known as patient-generated health data (PGHD) [16]. Assisted living technologies (known as “welfare technologies” in Norway) refer to technologies that augment an individual’s safety and security, including technology used for remote care. These technologies also manage and transfer PGHD. Assisted living technology, remote care, and disease management technologies have been adopted broadly in Norway and Canada. This adoption is expected to increase drastically in the coming years due to demographic shifts and health care resource constraints [17,18]. The 2 countries differ in geographical size and population, but they share a high degree of trust in public institutions and maintain predominantly publicly funded health care systems. Both also share a decentralization of health care delivery; in Canada, health care services are managed by 10 provincial health authorities and 3 territorial authorities, while in Norway, primary health care is distributed among 356 municipalities, while specialist health care is distributed among 4 regional health care authorities. Moreover, the populations of both countries demonstrate high levels of technological literacy, with an internet penetration rate of 99% in Norway and 92% in Canada [19]. Although technological literacy is high, cybersecurity still poses a challenge.

Cybersecurity can be understood as the “protection of computer systems and networks from attack by malicious actors that may result in unauthorized information disclosure, theft of, or damage to hardware, software, or data, as well as from the disruption or misdirection of the services they provide” [20]. In health care, the implications of cybersecurity incidents extend beyond simple data breaches to encompass critical consequences, potentially harming the health and safety of individuals and compromising PGHD [1,21]. The successful deployment of remote care solutions depends on several elements, including technology accessibility, technological literacy, financial resources, and health care delivery’s structural and organizational aspects [22-24]. It involves many stakeholders, including patients, clinicians, public and private health care organizations, technology vendors, and regulatory bodies [25-27]. The stakeholders involved in device use, operation, and management may hold different perceptions of cybersecurity risks.

Cybersecurity risk perception may differ between patients and health care workers; a patient using a type 1 diabetes (T1D) device may be more concerned about the immediate threat of denial of service impacting their insulin delivery, while a clinician might focus on the broader implications of compromised data integrity across both T1D devices and wearables for clinical decision-making. Cybersecurity risk perception encompasses concerns about threats and their potential impact on technologies, systems, and data, including incidents such as data breaches, attacks, and hacking, which can compromise confidentiality, integrity, availability, and functionality. Understanding these risks requires technical considerations and the social, cultural, and psychological factors that shape how individuals and organizations perceive cybersecurity threats [28,29].

Various disciplines, such as psychology, engineering, and safety science, have explored risk perception. Common theories in the risk perception field include the social amplification of risk framework and protection motivation theory [30-36]. Factors such as culture, optimism bias, personal fear, dread, familiarity, benefit, trust, and social trust have been explored in previous research on cybersecurity risk perception [30-33,37-45]. The Technology Adoption Model (TAM) and TAM2 are widely accepted in explaining why particular technologies are adopted [46-51]. Previous research highlights a lack of focus on human factors when assessing sociotechnical systems, especially within cybersecurity. A study found that technical factors often receive the most attention, whereas human factors related to awareness, privacy, and trust are frequently overlooked [52]. Addressing these human factors is crucial for a comprehensive understanding of cybersecurity risks in health care. Therefore, we adopted a cybersecurity approach to contextual factors, dividing them into human, organizational, and technological categories [53,54].

### Aim

This paper aims to investigate cybersecurity risk perception for assisted living technologies and wearables among stakeholders from Norway and British Columbia (BC), Canada, and identify contextual factors that shape these perceptions. We aim to explore differences in cybersecurity risk perceptions among different stakeholder groups for these technologies and identify the relevant human, organizational, and technological factors that impact these differences. However, due to ethical considerations, we do not compare survey data from the 2 geographical locations; instead, we rely on the interpretation of qualitative data from interviews to identify if differences in risk perception exist.

## Methods

### Study Design

This study performed an analysis among relevant stakeholders in Norway and BC, Canada, to explore cybersecurity risk perception and contextual factors associated with assisted living technologies. To simplify reporting, we refer to “cybersecurity risk perception” as “risk perception.” Our sampling approach in Norway was based on previous research on key stakeholders for assisted living technologies [23,55,56]. In Norway, we focused on 2 specific technologies, medicine dispensers and digital self-reporting forms, as these solutions have been widely adopted. We approached stakeholders to participate as survey respondents and as interviewees. In BC, these same technologies have not been as widely adopted. Hence, we examined other technologies that support an individual in managing their disease remotely: insulin pumps and continuous glucose monitoring (CGM) devices. These technologies are used as examples in the survey and interviews.

**Figure 1:**
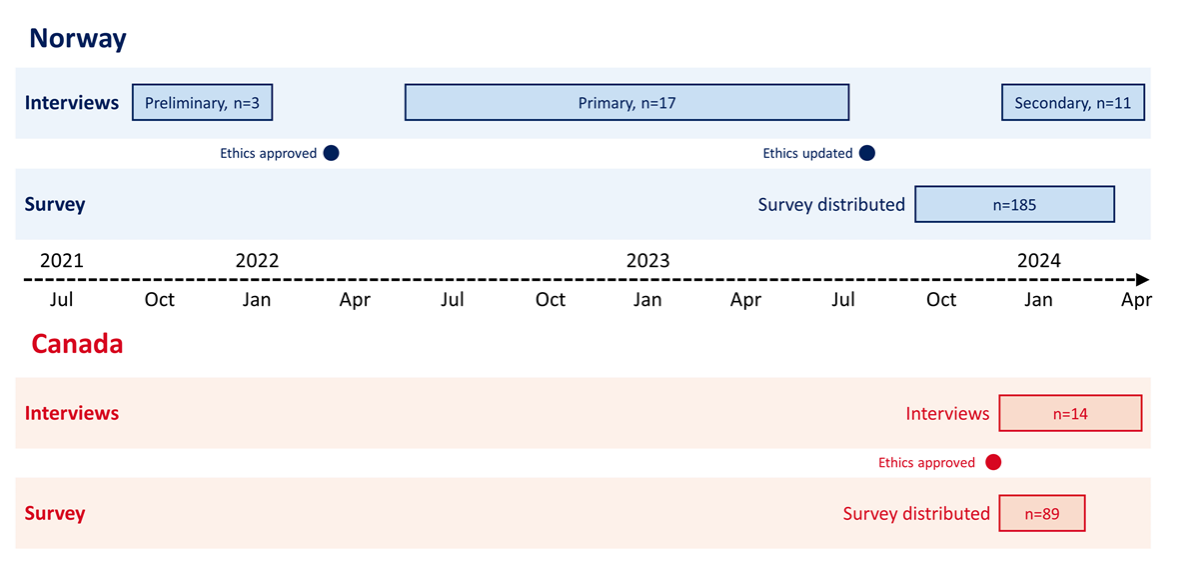
Study timeline showing study phases, data collection periods, and ethical approvals.

The survey was distributed in Norway from September 2023 to March 2024, and interviews were conducted from August 2021 to February 2024. Three preliminary interviews were conducted before ethical approval, which was permitted by Norwegian ethics rules but restricted the ability to gather any identifiable information. However, they enabled the gathering of key insights into the organization and implementation of assisted living technology in the Norwegian health care sector, which were instrumental in the subsequent research design. The survey was distributed in BC from December 2023 to February 2024, and interviews were conducted from December 2023 to April 2024 (Figure 1). This manuscript has been prepared following the COREQ (Consolidated Criteria for Reporting Qualitative Research) guidelines [57].

### Participants

Participants were health care workers, patients or device users, and experts involved in the development and implementation of assisted living technologies and wearables. The stakeholder groups enrolled varied slightly between the 2 countries. Specifically, Norwegian participants included health care workers in primary and specialist care, device vendors or industry professionals, health authorities, and cybersecurity experts. The participants from BC were patients (adults and adolescents), families of patients, clinicians or researchers using health care–related wearable technologies or data, biomedical engineers, hospital or research information technology (IT) professionals, and device vendors or industry professionals. We included adolescents to enrich the data material, as they represent a key user group for the T1D technology use case. Thus, it helped to offer a more comprehensive understanding of the cybersecurity risk affiliated with the technology across different demographics.

### Recruitment

The survey and interview respondents in Norway were gathered through an initial email to primary health care providers, specialist health care providers, health care authorities, technology vendors, and the expert network at the Norwegian University of Science and Technology. The survey was distributed by leaders in either primary or specialist health care organizations and a group dedicated to health care technologies in Norway on the social media platform Facebook (Meta Platforms, Inc.). In BC, we recruited adolescents, their parents, and other adults living with T1D using existing lists of participants who had given permission to be contacted for research and additional adult patients through a provincial portal for patient engagement and recruitment: REACHBC [58]. Health care clinicians, researchers, IT professionals, biomedical engineers, and device vendors/industry professionals were contacted via email and newsletters utilizing existing networks; participants belonging to health care institutions were approached via departmental email lists and research institute newsletters.

### Survey Instrument

The survey deployed in both countries was built on the Behavioral-Cognitive Internet Security Questionnaire (BCISQ), which uses a 5-point Likert scale to gather data on respondents’ behavior and awareness of using solutions based on information and communications technology [59]. Our survey consisted of 3 main parts: the first part gathered background information, including age, gender, and profession; the second part included the BCISQ; and the final part collected insights on risk perception for assisted living technologies or diabetes technology and smartwatches.

The BCISQ measures behavior and perception of information security risk when using the internet in 2 ways: first, by having participants assess and respond to various “risky” situations, and subsequently, by simulating a scenario that asks for the respondent’s most commonly used password question. In Norway, the simulation was presented through the open-ended question: “To check the quality of your password, write your most used password.” This could not be replicated in BC due to ethical considerations and concerns by institutional research privacy leaders. Instead, we posed the question, “If we asked you to reveal your most used password to evaluate the security of your password, would you reveal it?” with the answer option “yes/no”. Two subscales measure the cognitive dimension; the first scale captures an evaluation of information security risk, utilizing the term “likelihood” as per Adams [60] to understand how users evaluate the probability of cyber risk, while the second subscale captures how important users evaluated security mechanisms in managing these risks, following the approach of Velkie and Solic [59] who evaluated the individual’s assessment of risk and perceived importance of security mechanisms. For example, the first scale contained questions to evaluate risk, such as “How would you rate the likelihood of someone stealing your identity on the internet (some examples of services that use your personal identity include online banking, social media, and e-mail)?” with options ranging from “very unlikely” to “very likely”, while importance was captured by a direct question, such as “How would you rate the importance of updating your smartphone or laptop with the latest software?”, with options on a scale of “not very important” to “very important.” [59,60].

We extended the survey to encompass questions related to assisted living technologies or diabetes technology and smartwatches to enhance our understanding of risk perceptions associated with specific technologies. Smartwatches were intended to target a technology widely recognized by the general population yet not primarily serving a dedicated health function. This allowed us to explore if risk perception differs between assisted living and diabetes technologies versus smartwatches. The survey sought to capture respondents’ evaluations of the likelihood and impact (consequence) of incidents compromising confidentiality, integrity, and availability. Continuing the structure of the BCISQ, responses were collected on the same 5-point Likert scale.

Before deploying the surveys in Norway and BC, we conducted preliminary tests with researchers, mainly from the health care field. These tests led to minor modifications in the question structure and sequence, ensuring clarity and relevance in both contexts. The survey was distributed in Norwegian in Norway and in English in BC. The surveys can be found in Multimedia Appendix 1 (English version) and Multimedia Appendix 2 (Norwegian version).

### Interviews

A semistructured interview approach was adopted, and interview guides were prepared in advance. These aimed at gathering comprehensive data on key areas: (1) understanding of assisted living technologies or diabetes technologies; (2) cybersecurity risk understanding; (3) cybersecurity threats and vulnerability specific to these technologies; (4) behavior, attitude, and awareness; and (5) organizational and management practices for technology and security. Because of the varied knowledge and characteristics of the stakeholders, we developed customized interview guides. In Norway, interview guides were tailored to each stakeholder group to reflect their unique perspectives and roles. In BC, an initial interview guide was developed and adjusted according to the specific stakeholder being interviewed. Primary themes were consistent, yet the flexibility in the interview process allowed for follow-up on specific topics based on the interviewee’s responses. For instance, health care workers familiar with one of the technologies were asked more detailed follow-up questions regarding their risk perceptions of that technology. Conversely, for those less familiar with specific technologies, discussions were generally focused on risk perceptions of assisted living technologies or smartwatches. The interview guides are attached in Multimedia Appendix 3 (English version) and Multimedia Appendix 4 (Norwegian version).

### Analysis

Descriptive statistics were applied to the survey data in both data sets to summarize participants’ characteristics and responses to questions related to the BCISQ (behavior and attitude) and risk perception. Variability was assessed using Cronbach α, indicating the internal consistency of the items. A significant challenge in our analysis was comparing the 2 data sets subject to different privacy regulations. The Norwegian data were stored in Norway, and data transfer to other countries, especially outside the European Union, was prohibited. Similarly, the BC data were stored in BC, and transferring data outside Canada was prohibited. The data sets were analyzed separately to overcome these challenges and account for variation in the specific stakeholder groups participating in the 2 countries. Subsequently, the results of the aggregated data were subjected to further analysis and comparison. We used R statistical software (version 4.3.2; R Foundation for Statistical Computing) and Excel (Microsoft Corporation) to analyze the quantitative data.

Interviews were transcribed and verified against the recordings, followed by analysis using NVivo (Lumivero) for thematic analysis. We used a stepwise deductive-inductive approach. This process began with establishing codes and progressed to linking codes via axial coding, facilitating the identification of relationships between codes [61]. Subsequently, we developed categories encompassing multiple codes, enabling the identification of key concepts and themes. The stepwise inductive-deductive method allowed us to continuously refine our understanding of empirical findings supported by theory [61]. This approach also aligns with the suggested approach for conducting thematic analysis by Braun and Clarke [62]. Throughout the process, the researchers discussed recurring themes to enhance the consistency of the analysis. When analyzing the qualitative data, we completed separate analyses to prevent data transfer and then aggregated and interpreted data on themes and recurring topics between both data sets.

### Ethical Considerations

Ethical approval in Norway was obtained from the Norwegian Agency for Shared Services in Education and Research (606692) on March 21, 2022, with updated approval on August 15, 2023, to reflect the data from BC. Ethical approval in BC was granted by the University of British Columbia/Children’s and Women’s Health Centre of British Columbia Research Ethics Board (H23-01987; date of approval 2023-11-27; principal investigator: MG). Implied informed consent was used for surveys, while electronic consent was used for interviews.

Written informed consent was obtained for all interviews. Before each interview, participants were emailed a consent form. As a result of different privacy legislation, the forms were collected in different ways. We used the Research Electronic Data Capture (REDCap) electronic consent framework for BC, while in Norway, the forms were distributed and collected via email, with participants signing and returning the consent form [63].

Privacy and confidentiality protection were priorities in this study. Data were analyzed once samples were deidentified. Norwegian data were stored on an encrypted device with password protection, and the tool used to collect survey data did not collect metadata or IP addresses to prevent the identification of human participants. In BC, data were collected through REDCap, which also did not record IP addresses. Extracted data were stored on research institute network drives, with access restricted to research team members.

Norwegian research participants received no compensation for their participation. In BC, survey participants were entered into a raffle for approximately US $7.50 gift cards, while all the interview participants received an approximately US $15 gift card.

## Results

### Participation Overview

We received 274 survey responses (Norway, n=185; BC n=89). Participants were predominantly female (139/185, 75.1%, in Norway and 57/89, 64%, in BC) and most respondents were between 21 and 56 years old; 45 interviews were completed (Norway n=31, BC n=14). The median age category was 46-50 years in Norway and 41-45 years in BC (Table 1).

**Table 1:** Overview of the data sample.

Demographics			Survey					Interview		
			Norway (n=185)		British Columbia (n=89)		Norway (n=31)			British Columbia (n=14)
**Age (years) group, n (%)**										
	15-20	N/A^a^		5 (5.6)		N/A			N/A	
	21-25	6 (3.2)		12 (13.5)		N/A			N/A	
	26-30	15 (8.1)		6 (6.7)		N/A			N/A	
	31-35	23 (12.4)		11 (12.4)		N/A			N/A	
	36-40	21 (11.4)		13 (14.6)		N/A			N/A	
	41-45	29 (15.7)		6 (6.7)		N/A			N/A	
	46-50	23 (12.4)		4 (4.5)		N/A			N/A	
	51-55	13 (7.0)		11 (12.4)		N/A			N/A	
	56-60	27 (14.6)		8 (9.0)		N/A			N/A	
	61-65	21 (11.4)		7 (7.9)		N/A			N/A	
	66-70	3 (1.6)		2 (2.2)		N/A			N/A	
	71-75	4 (2.2)		2 (2.2)		N/A			N/A	
	76-80	0 (0)		1 (1.1)		N/A			N/A	
	Missing	N/A		1 (1.1)		N/A			N/A	
**Gender, n (%)**										
	Male	45 (24.3)		26 (29.2)		15 (48.4)			9 (64.3)	
	Female	138 (74.6)		57 (64.0)		16 (51.6)			5 (35.7)	
	Prefer not to answer/others	2 (1.1)		6 (6.7)		N/A			N/A	
**Participant type, n (%)**										
	Health care worker	171 (92.4)		8 (9.0)		20 (64.5)			4 (28.6)	
	Industry professional/vendor	N/A		N/A		6 (19.4)			2 (14.3)	
	Health authority personnel	N/A		N/A		3 (9.7)			N/A	
	Patient (adult or adolescent) or family member of the patient	N/A		69 (77.5)		N/A			7 (50.0)	
	Biomedical engineer, hospital, or research information technology professional	N/A		12 (13.5)		N/A			1 (7.1)	
	Cybersecurity expert	N/A		N/A		2 (6.5)			N/A	
	Others	14 (7.6)		N/A		N/A			N/A	

^a^N/A: not applicable.

### BCISQ

BCISQ data demonstrated that both respondent groups assessed their behavior as low-risk (Table 2). The groups had a median score of 1 and 1.1 (never). The range of the group demonstrates that few of the respondents ever engage in “risky” behavior, regardless of country. On the cognitive likelihood (risk) scale, both groups demonstrated a neutral to unlikely understanding of the occurrence of an undesired event. However, the range was wider in Norway (range 1.6) versus BC (range 1.0). Norway had a median score of 4 on the cognitive importance scale, while BC had a 3.8 median score.

The last instrument of the BCISQ was the behavioral simulation. In total, 46 out of 185 (24.9%) Norwegian respondents answered the question: 17 out of 185 (9.2%) were deemed to have provided a possible actual password based on password requirements (capital letters, special characters, and password length), while the remaining 29 provided responses that were not deemed to be their actual password. Of the BC respondents, 16 out of 89 (18%) answered that they would provide their password. Moreover, when asked about password strategy, the majority in both groups reported that they only implemented minor password changes when changing passwords (94/185, 50.8%, in Norway and 45/89, 51%, in BC).

**Table 2:** BCISQ^a^ subscale data split by region.

Region	Norway, median (IQR)	British Columbia median (IQR)
BCISQ subscales		
Risky behavior self-assessment scale	1.0 (1.0-1.0)	1.1 (1.0, 1.2)
Cognitive likelihood (risk) scale	3.0 (2.2-3.8)	2.8 (2.2, 3.2)
Cognitive importance scale	4.0 (3.25-5.0)	3.8 (3.0, 4.2)

^a^BCISQ: Behavioral-Cognitive Internet Security Questionnaire.

### Reliability Analysis

The Norwegian data sample demonstrated internal consistency with Cronbach α, which was above the acceptable rate (0.7) on the overall questionnaire and the cognitive likelihood and importance scales, but lower on the behavioral scale. The BC responses demonstrated acceptable internal consistency overall and for the cognitive likelihood and importance scales, but the risky behavior self-assessment measurement was not within the acceptable range (Table 3).

**Table 3:** Cronbach α for the whole BCISQ^a^ instrument and each submeasurement, split by region.

Behavioral-cognitive scale	Norway	British Columbia
BCISQ (total)	0.767	0.718
Risky behavior self-assessment scale	0.643	0.446
Cognitive likelihood (risk) scale	0.882	0.745
Cognitive importance scale	0.752	0.746

^a^BCISQ: Behavioral-Cognitive Internet Security Questionnaire.

### Risk Perception for Assisted Living Technologies and Diabetes Technologies

**Figure 2:**
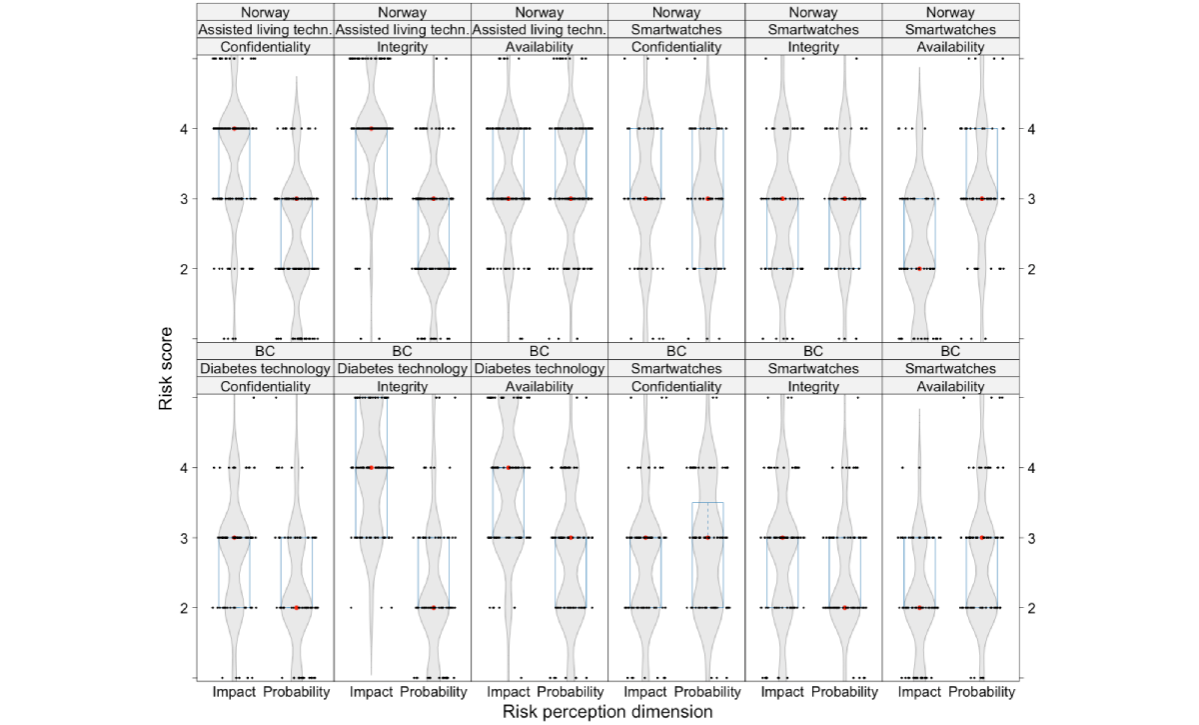
Risk perception related to the consequences (impact) and likelihood (probability) of cybersecurity-related issues, split by risk dimension, technology type, and location. Data are shown as violin plots (in grey) showing the data distributions, overlaid by boxplots (blue box denoting the first and third quartile, with the red dot denoting the median) with the raw data superimposed as black dots. BC: British Columbia.

For assisted living and diabetes technologies, the likelihood (probability) of confidentiality, integrity, or availability being compromised had a median score of 2 (unlikely) to 3 (neutral) among all respondents (Figure 2).

Because of a survey logic error, which resulted in optional risk perception questions, the Norwegian sample was limited to 147 out of 185 (79.5%) participants. The range for availability was slightly higher for assisted living technologies (IQR 3-4). Results for consequences (impact) were evaluated higher, between 3 (moderate) and 4 (major), indicating a more serious evaluation of consequence. For diabetes technologies, the range for integrity had the highest consequence, between 3 (major) and 5 (catastrophic). The Cronbach α was 0.679 for the Norwegian data sample and 0.664 for the BC sample.

### Risk Perceptions for Smartwatches

For smartwatches, the likelihood (probability) of confidentiality, integrity, or availability being compromised had a median in both populations between 2 (unlikely) and 3 (neutral), with most results at 3 (neutral; Figure 2). The IQR was between 2 and 3 for all measurements. The results for consequences (impact) were between 2 (minor) and 3 (moderate). As a result of a survey logic error, which resulted in optional risk perception questions, the Norwegian sample was limited to 65 out of 185 (35.1%) participants. For the smartwatch risk perception measurement in Norway, Cronbach α was 0.812, and for BC, it was 0.694.

### Interview Findings

We conducted 45 interviews (Norway n=31, BC n=14), involving 25 females (Norway n=17, BC n=8) and 23 males (Norway n=18, BC n=5). The initial coding process resulted in 72 codes for Norway and 61 for BC. After aggregating into categories encompassing all codes, we had 10 categories for BC and 10 categories for Norway, which mapped to 3 overarching themes derived from the theory on contextual factors in cybersecurity: organizational, technological, and human factors. As some categories overlapped, this resulted in 14 themes (Textbox 1).

**Contextual factor category and themes and subthemes.**

**Organizational factor**

- Health care organization, systems, and processes
- Regulations, compliance, and ethics
- Risk management, safety, and privacy
- Vendor and third-party relationships
- Goals and objectives
- Cost and accessibility

**Technological factor**

- Technology, implementation, and effect
- Security and privacy
- Technology perception and data sharing
- Device reliability, criticality, and impact

**Human factor**

- Stakeholder perspective and responsibility
- Knowledge, competence, and awareness
- Trust
- Benefit and convenience

### Organizational Factors

Contextual factors encompass factors specific to organizations, such as organizational structure, culture, and roles and responsibilities, which affect how risks are perceived and managed. In the analysis, we identified themes that included several contextual factors (Table 4).

**Table 4:** Organizational factors identified from interviews.

Theme and factor			Explanation		Example quotes from participants in Norway (N) and Canada (C)
**Health care organization, systems, and processes**					
	Management of security and technology	This refers to how cybersecurity and technologies are managed within an organization to protect the PGHD^a^ and medical devices. This influence risk perception through organizations capabilities and security practices.		“We talked about silos. Health is no longer just health; it is IT, technological, education. We are so dependent on that collaboration and the flow between them, especially when we start with technology and undergo this digital transformation, it is difficult to sit in separate silos. It is all so interconnected.” [N10] “When it (devices) is kept within the hospital, it is kept there, but when you take out the solutions, they are exposed to new risks, and you have to go slowly to make it safe.” [N16]	
	Organizational structure	Organizational structure refers to how different health care organizations are structured, which can impact the abilities and clarity of security management.		“(...) we have different layers. [In] primary care, specialist care, and tertiary care, on the other hand, we also see how the organizational aspects are managed and controlled by different authorities, which makes the issue [cybersecurity risk] even more difficult to deal with.” [N31] “Specialist health care is much more competent and centralized, also in terms of competence. I experience that generally from the municipalities, who do not have a lot of competence. They place a lot of trust in us.” [N29]	
	Understanding of responsibility	Refers to how responsibility for security is organized and shared. When responsibility is defined and understood, it indicates that risks are addressed and technologies are safeguarded.		“One important thing in Norwegian health care is that there are interfaces that will be challenging and have not been addressed. (...) Where is the interface, and how should it be managed? Where should the data go, and who is responsible for the security?” [N15] “(...) like any personal information that I have responsibility for, but even that is sometimes in conflict with the guidance we get from the hospital or university. Then that creates like this other layer of, I don’t know if you want to call it moral distress, I mean, like who am I supposed to listen to?” [C6]	
**Vendor and third-party relationships**					
	Vendor expectations	The security expectations of vendors and the belief that vendors have the abilities and capabilities to meet these are also important.		“(vendors) must be capable of offering solutions that do not fall apart once you start picking (investigating) them.” [N30] “So I trust the Medical Device Company more both in terms of reliability of the device, but also the in terms of it’s data security, right?” [C14]	
	Dependencies	Dependencies can heighten or lower risk perception based on the perceived criticality of dependency. The more dependencies, the more complex a system is, which can introduce vulnerabilities and potential risks.		“No matter if it is in the health care sector or not, long vendor supply chains are a challenge. One thing is development and drift. Development with a long supply chain can [mean that] those who develop are too far from those using them. There is too big [of a] distance.” [N17] “There have not been any incidents yet at any of our vendors, but there are no guarantees. So that is a major concern. We are very dependent on these vendors.” [N25]	
	Third parties	These refer to external dependencies handling sensitive data or critical operations/components.		“Of our product, maybe 60-70% of our products are from third parties, such as equipment and software.” [N24]	

^a^PGHD: patient-generated health data.

### Technological Factors

Technological factors include how technologies are implemented, which security mechanisms are integrated, and their functionality. This category refers to the characteristics and capabilities of the technology and how the respondents perceived cybersecurity risk based on their understanding of different technological and system-related factors (Table 5).

**Table 5:** Technological factors identified from interviews.

Theme and factor		Explanation	Example quotes from participants in Norway (N) and Canada (C)
**Technology, implementation, and effect**			
	Technological dependencies	Describe the perceived reliance on technologies outside of the actor’s control, which can heighten risk exposure.	“...there are so many dependencies on the clients that we can't upgrade quickly enough – it takes a lot of time and a lot of money.” [N15] “We are increasing the digital footprint – the more sensors we get and the more systems we push out will increase the risk that someone can reach something.” [N16]
	Security mechanisms	The tools implemented to protect data and technology and the confidence placed in their effectiveness impact risk management capabilities and perceived risk.	“I wouldn't say like a hack, but it's really easy to get information. So you really got to take safety measures if you want to not get your identity stolen or your accounts hacked, and that kind of stuff.” [C4] “Data, accuracy, and security. but you know, like you, you pay a price for it, right? Because devices are very expensive.” [C14]
**Device reliability, functionality, and criticality**			
	Reliability	Refers to the perceived trustworthiness of the device, the consistent performance of technology, and the absence of system failures.	“I’m always a little bit sceptical about, you know, how reliable that data is, and how helpful it is for them to be tracking sometimes, at this micro level.” [C6] “(...) Then I would have to choose accuracy, because that’s what I need to know, that what I’m I need to believe that what I'm seeing is real right, and it reflects the state of the patient in front of me. Right?” [C13]
	Functionality	The different tasks a system or technology performs affect the user’s utility of the technology	“(worst case)(...) if it just stopped working altogether” [C3] “I was kind of scared at the beginning. But I'm like, okay, whatever, as long as it works, it works, right?” [C12]
	Criticality	This refers to the perceived importance of technology in health care, where failure can result in severe impacts, which would increase perceived risk.	“Oh, what if you know bad actors kind of interject and you know they give like extra bolus, like, I don't know. Maybe some people might say like, 'Oh, you’re, you know, like creating this novel story,' right? But who knows? Right? So those kinds of like it's fear always back up my mind. Like I said. I don't use my phone to bolus.” [C12] “I have been concerned, particularly when thinking about what I talked about before, like a looped system where the pump can control, or you can control both the blood glucose monitor and the pump with the same system.” [C4]
**Benefit and convenience**			
	Convenience	The accessibility and ease of use of technology.	“Because anytime you add encryption, you add security, [but] you added a bit more inconvenience.” [C2]
	Understanding of the value of data	Understanding of the importance and sensitivity of data. The sensitivity level of data may affect the risks associated with it.	“If they got a hold of your medical data, could they like - what would they do with it? I'm not aware of, like, what? Why would someone take that, and what to do with it unless it was going to be to blackmail you?" [C11] “I feel like I expect that my data will be safe and protected.” [C4]
**Technology perception and data sharing**			
	Data sharing	Refers to the processes and procedures for data sharing and the attitude toward data sharing.	“There is a huge security risk when you start talking about data flow and data sharing, that you have so many instances that you must have security at each instance.” [N15] “I think it’s continuous. I don't have to like even I remember I had to delete the app (...) But I it seems like it has my information, and it continues to do so.” [C12]

### Human Factors

The concept of human factors refers to the physiological and behavioral elements that influence how cybersecurity and risks are perceived for systems and technology (Table 6).

**Table 6:** Human factors identified from interviews.

Theme and factor		Explanation	Example quotes from participants in Norway (N) and Canada (C)
**Knowledge, competence, and awareness**			
	Knowledge	The level of understanding either an individual or organization has about cybersecurity, threats, and risks.	“If we had more knowledge/competence, then maybe the problem would disappear.” [N1] “I think about this stuff all the time, and I worry about it a lot. In part because I feel like I don't understand it enough.” [C6]
	Competence	Cybersecurity refers to the skills and abilities one has in cybersecurity, enabling the individual to understand and manage cybersecurity and how cybersecurity incidents are managed.	“I think everyone understands that this (cybersecurity) is important; however, there is a lack of competence.” [N24] “No, I believe it is humans. It is without a doubt that and lack of competence.” [N29]
	Awareness	The degree to which one recognizes the importance of cybersecurity and affiliated risk and how the individual behaves based on this.	“It’s not a question of if (an attack will occur), but when.” [N30] “I don’t know if I’ve ever thought about it being altered like sort of like, nefariously or maliciously.” [C6]
**Stakeholder perspective and responsibility**			
	Fear and severity of consequence	The dangers or severity affiliated with the potential consequences of a cybersecurity incident.	“I just sleep through my low blood sugars. That's scary. I might not be able to wake up in the morning right? (...) Usually the pump usually peeps so I'm usually like be able to treat myself, but at that time I don't know what happened, and so I was sleeping through (...).” [C12] “If you think about the worst-case scenario, it could be that users are sitting at home who believe these alarms work, and they become ill for some reason and trigger this alarm, but it doesn't work as they expect it to. So, in the worst case, it could result in death.” [N3]
	Potential risk scenarios and concerns	The perceived incidents that could occur.	“I don’t know what the feasibility of is like somebody hacking someone's insulin pump and overdosing or underdosing (...) because I suppose it would be hacking the CGM and telling the insulin pump to do so like.” [C15] “A data breach is very serious because it involves very sensitive data for patients, and we have seen that it is very serious for the municipalities. It could greatly affect what people think about our organization's solutions, so I think that's actually the maximum consequence.” [N24]
**Trust**			
	Trust in technology	The confidence placed in the technology to be reliable and secure.	“I think my trust. Yes. Although it's really a case of going from a state of no knowledge or no experience, and therefore may be no trust to yeah using this as a reliable trend monitor and saying, 'Yeah, this is fine,' you know, and if I need to verify it, I will do.” [C13] “I'm just hoping and trusting that the technology, the companies making these products. They have some kind of security at their end.” [C9]
	Trust in institutions/in organizations	The confidence placed in institutions or organizations to be reliable and secure in their management of systems, technology, and data.	“I would say I trust them, and I trust that they keep my data private and they don't share it with anybody (...).” [C3] “The fact that one (municipalities) just trusts the vendor (blindly) is not very good.” [N29] “(...) you are heavily influenced by the atmosphere of trust, so people trust each other.” [N31]
**Benefit and convenience**			
	Perceived benefit	The advantages related to the use of technology or specific devices.	“Because I know that it's helped my management a lot, so I think I would probably take risks for that.” [C11] “Just think, what the devices bring to the table is so life-changing that it would have to be really a risk that had it had been clearly identified that it was super high for anybody to really take much notice.” [C9]
	Cost/benefit	The trade-offs made between risk and benefits.	“...it’s really a balance between how the convenience of use and the level of protection you can provide” [C2]. “So you know, like most decisions in medicine, are sort of either risk, benefit, or cost-benefit equation (...) where better is defined as whatever better is defined as, right? It may be a trade-off right?” [C13]

## Discussion

### Principal Findings

For assisted living technologies, cybersecurity risk was perceived as reasonably low. Yet, there were variations in how confidentiality, integrity, and availability were assessed between technologies. Findings from the BCISQ demonstrated similar behavior and awareness among respondents, but risk perceptions for different technologies varied. For assisted living technology, integrity and confidentiality were prioritized, whereas, for diabetes technology, availability was viewed as more critical. Moreover, our survey and interview findings indicated that risk perception is stronger toward assisted living and diabetes technologies than smartwatches. This nuance was further explored in interviews, where both groups acknowledged availability as a crucial aspect of device functionality. Although few respondents considered data or device manipulation likely, they expressed significant concern about the potential consequences if such events occurred. Risk perceptions of the respondents are significantly affected by contextual factors in the area of human factors, namely, knowledge, competence, familiarity, dread/security of consequence, perceived benefit, and trust. The technological factor of “functionality and criticality” was also found to affect risk perception, as demonstrated in the differences between “high-risk” and “low-risk” devices. These are the factors evaluated to have the greatest implications for risk perception. Organizational factors such as health care sector organization were identified to have an effect, but based on the interviews, less than the others.

### Risky Behavior and Information Security Awareness

The results from the BCISQ demonstrated that our respondents assessed their behavior as secure, with a more unlikely than likely chance of being exploited. Security measures were evaluated as high in importance, which indicates a strong awareness among respondents. There were only minor variations in the data between the included participants, indicating that respondents have similar behavior and awareness about cybersecurity. However, a discrepancy was identified between how respondents self-assessed their behavior and how they behaved: 17 out of 185 (9.2%) Norwegian respondents and 16 out of 89 (18%) BC respondents were willing to engage in risky behavior by revealing their passwords to check password quality. In addition, 94 out of 185 (50.8%) Norwegian respondents and 45 out of 89 (51%) respondents reused passwords, which is viewed as risky behavior and bad practices within cybersecurity.

### Comparison With Prior Work

#### Organizational Factors

In our study, factors such as organizational structure, roles and responsibilities, and expectations toward vendors influenced how risks were perceived. This is supported by previous research, which found that organizational factors can contribute to increase (amplify) or decrease (attenuate) perception of risks [35]. Moreover, previous research has found that cross-cultural differences can affect risk perceptions [34,35]. Our survey suggested minimal differences among respondents from Norway and BC in behavior, awareness, and view of risks for health care technology and smartwatches. Similarly, our interviews found similarities in risk perceptions between the participants, indicating that region (cross-cultural differences) has a minimal effect on risk perception in this case [44,45].

#### Technological Factors

We found similar understandings of the risks of assisted living and diabetes technologies based on the consequences if confidentiality, integrity, or availability were compromised. A minor difference was found where availability risks were evaluated higher in consequence for insulin pumps and CGMs (4=major), while integrity and confidentiality risks were evaluated higher for medicine dispensers and digital self-reporting (4=major).

Smartwatches were included to investigate if respondents perceived risks differently for “low-risk” compared with “high-risk” devices, as risk perception has previously been connected to the severity and fear of consequences [39,42,44,48,64,65]. Our survey identified differences in risk perception, where “high-risk” devices included assisted living technologies and diabetes technologies, and “low-risk” included smartwatches. The probability and consequences were evaluated higher for “high-risk” devices than “low-risk” devices (Figure 2). In interviews, the perceived consequences of a closed-loop insulin control system were viewed as potentially fatal, which was a consideration for adopting such technologies. Manipulation of data, compromising data integrity, could lead to devastating consequences, such as wrong insulin/medication dosage. This indicates that the function and criticality of the device affect risk perception and technology adoption, which aligns with previous research [39,64,66,67].

#### Human Factors

Previous research has demonstrated that human factors such as knowledge, behavior, and emotions are essential for understanding cybersecurity risk [52]. Risk perception theory highlights that understanding, competence, and familiarity with both hazards and risks affect risk perception. Yet, the TAM views familiarity as an enabler for technology adoption [29,44,47,50,51,65,66]. Findings from our study agree with previous studies, where increased familiarity and knowledge of a technology resulted in a change in risk perception. Thus, the perceived severity of consequences affected risk perception, agreeing with previous research emphasizing that the severity level will increase or decrease risk perception [30-32,45]. The concept of dread has been investigated thoroughly within risk perception theories [66,68]. Dread associated with potential harm affects risk perception: in both interviews and our survey, consequences were evaluated as more severe, with potentially fatal consequences. Within TAM and risk perception studies, it has been found that perceived usefulness is important for technology adoption and that the benefits of technology lead to greater risk acceptance, followed by a lower risk perception [42-44,67]. Similarly, we found that respondents were willing to accept risk up to a certain level due to the perceived benefits of the devices. This indicates that convenience and benefit also shaped how risks were perceived, resulting in a lower risk perception.

The implication of trust on risk perception has been debated in previous work [40]. In our study, trust was an essential factor influencing risk perception. Our respondents expressed trust in health care institutions and medical device companies to ensure that technology is secure, previously conceptualized as social trust [42]. Trust is also placed on individuals, whereas if a user trusts others interacting with technologies, risks are perceived to be lower [43,44]. Lastly, there is also trust dedicated to technologies, as respondents perceived these as trustworthy due to a lack of incidents, which resulted in lower perceived risk for assisted living technologies and diabetes technologies.

### Limitations

The starting point of this study was to investigate cybersecurity perceptions among stakeholders in 2 different countries. The first limitation concerns the inherent differences between these 2 countries. Although we recognize these differences, we can compare risk perceptions due to a focus on technologies deployed in the health care sector. We recognize differences between users of assisted living technologies and diabetes technologies; however, the findings provide valuable insight into cybersecurity risk perception. A limitation in the Norwegian data sample is the exclusion of patients, which should be addressed in future studies to provide a more comprehensive perspective. In continuing this research, including patients as part of the Norwegian sample would be advisable to capture their concerns about cybersecurity risks.

Next, due to a survey logic error that resulted in optional risk perception questions, the Norwegian sample was limited to 147 out of 185 (79.5%) participants for assisted living technologies and 65 out of 185 (35.1%) participants for smartwatches. This significantly reduces the power of the Norwegian sample in this dimension; nevertheless, we believe the observations still provide valuable insights.

Expanding the sample to include a wide range of patients in both countries would also allow a deeper exploration of how patient-specific factors, such as age, condition, or digital literacy, influence risk perception. Further, women are overrepresented in both samples (139/185, 75.1%, for Norway and 57/89, 64%, for BC). Although a more equal gender distribution would be desirable, it reflects the workforce composition in the health care sector in both regions [69,70]. Future research should aim for equal gender representation to ensure that one gender’s perspectives do not disproportionately shape the findings. Recruitment strategies could be adjusted to target more men or other underrepresented groups in the sample. Additionally, we did not perform a subgroup analysis by age category to explore if age is a factor that can explain some of the variability observed in risk perception, which should also be considered in future work, including more diverse populations and larger sample sizes.

Additionally, we could not combine the data sets for further statistical analysis, which restricts the depth of our analysis. The technologies used as examples in the study (assisted living or diabetes technology) serve different purposes and groups, which may lead to varying risk perceptions. This variability must be considered when interpreting the results, as it may influence stakeholders’ views on risk differently. Future research should aim to collect a larger sample, possibly through collaborations with multiple health care institutions. Additionally, future studies could consider longitudinal designs to identify changes in risk perceptions over time.

This study did not perform a subgroup analysis of survey responses by participant type, such as patients and clinicians. However, differences among patients and clinicians were observed in the interviews and, therefore, included in the discussion. Future research should investigate the effects of participant type in interpreting the survey data to more comprehensively explore the potential differences in risk perception by participant type or organizational role.

Next, the first author (AS) conducted the thematic analysis of interviews. This approach introduces the potential for bias, as a single researcher’s perspective can influence the interpretation of qualitative data. To mitigate this, the themes and key findings were discussed among the researchers. Future studies should aim for multiple researchers to engage in the thematic analysis process, ensuring a more balanced analysis. Furthermore, the reliance on self-reported data from surveys and interviews inherently carries the risk of response bias, where participants might provide socially desirable answers or have recall bias.

### Suggestions for Further Research

The BC cohort should be used to investigate differences between participant groups and user characteristics further. Including patients from the Norwegian population would also be interesting, as this could provide a more comprehensive understanding of risk perceptions and enable the comparison between patients in Norway and BC. A comparative analysis of Norwegian and BC patient populations could reveal how factors such as health care infrastructure, cultural attitudes, and patient demographics influence cybersecurity risk perception. This broader understanding could also inform more tailored approaches to risk communication and management in different health care contexts.

This study has examined cybersecurity risk perception related to specific individual health devices, yet they are often combined—a technological complexity that should be explored further. Further research should explore these complexities by investigating how interoperable systems manage cybersecurity risks, such as CGMs and insulin pumps working in tandem. This would involve assessing the technical vulnerabilities introduced by their interaction and understanding how users perceive or experience those risks in real-world settings. It may also be worthwhile to explore whether various vendor or operating system considerations (eg, Android vs iOS for smartphones) impact users’ risk perception.

Another promising direction would be to combine technological analysis of systems with human and behavioral studies, providing a comprehensive system-level perspective on cybersecurity risks in health care. Such research could offer insights into balancing cybersecurity’s technical aspects with user experiences, ultimately contributing to more secure health care technology ecosystems. In addition, future studies should incorporate mixed method approaches, such as triangulating self-reported data with observational data or objective measures (eg, usage logs or system performance data), to validate and complement the findings. This would provide a more comprehensive understanding of risk perceptions and reduce the potential bias in self-reporting.

### Practical Recommendations

Implementing technology is not straightforward, and one could argue it is even more challenging in health care due to patient safety and security considerations. Ensuring transparency about potential risks and threats is critical for fostering trust, as subjective perceptions of risk vary among interested parties, as identified in this study. For instance, when rolling out new devices such as closed-loop systems for diabetes management (T1D), health care workers and technology developers could hold regular informational sessions or consultations directed toward both care receivers (patients and end-users) and caregivers. Based on the identified needs, one could focus on explaining the security features of and fail-safe mechanisms implemented in these systems. Enabling ongoing risk communication could help reduce skepticism and perceived risk.

Moreover, a structured risk management process, including regularly updated risk and threat assessments, is essential. The study found that respondents were generally willing to accept a certain degree of risk when the perceived benefits and convenience of their device outweighed the potential risks. The balance between risk and benefit/convenience is a crucial driver of technology acceptance. For example, although users may have concerns about data privacy when using wearables, many still choose to use them because of the convenience and value of the health insights these devices provide—such as real-time glucose monitoring or medication assistance—as these are essential in managing their health.

In addition, strengthening the knowledge and competence of all relevant parties—developers, care receivers, and health care workers—is essential, as cybersecurity risks and threats can be challenging to understand, especially for novices. Awareness is a security measure that can mitigate risks derived from humans’ interaction with technology, and education is another area that should not be neglected to increase cybersecurity understanding. Practical steps such as security awareness programs could be integrated into the onboarding process for new technologies. For example, when introducing T1D technology (insulin pump or CGM) or assisted living technology (digital medication dispenser or digital self-reporting forms) to new users, as a part of the adoption process, one could introduce training sessions that cover key security areas such as password management, recognizing phishing attempts, and fail-safe mechanisms of the platforms. Such initiatives should be developed in close cooperation with the developers and vendors of the technology, as they often hold more detailed information about the system’s security features.

### Conclusions

We found minimal behavior and risk perception differences among the participants from Norway and BC. Human factors and device functionality were most influential in shaping cybersecurity risk perceptions. Considering the expected reliance on assisted living technologies and remote care solutions for disease management in the coming years, insight into risk perceptions can assist in reducing fears among relevant parties so that technologies can be adopted more broadly. Understanding risk perceptions can strengthen risk management and awareness, and understanding how to develop mitigating strategies to improve cybersecurity risk management is essential. Different inhibitors and drivers exist in technology adoption, so it is essential to understand the needs and capabilities of specific technology users. Building competence, knowledge, and trust can be essential steps in this endeavor. For future technological innovation and development, security should be considered as a prerequisite rather than an afterthought—it should be a license to operate.

## Appendix

Multimedia Appendix 1 British Columbia survey questionnaire.

Multimedia Appendix 2 Norway survey questionnaire (Norwegian).

Multimedia Appendix 3 Interview guide (British Columbia, Canada).

Multimedia Appendix 4 Interview guide (Norway; in Norwegian).

## Abbreviations

BC: British Columbia
BCISQ: Behavioral-Cognitive Internet Security Questionnaire
CGM: continuous glucose monitoring
COREQ: Consolidated Criteria for Reporting Qualitative Research
IT: information technology
PGHD: patient-generated health data
REDCap: Research Electronic Data Capture
T1D: type 1 diabetes
TAM: Technology Adoption Model

## Data Availability

Some data are available. Ethical approval in BC permits us to share deidentified survey data with other researchers upon request from the corresponding author, but not the interview transcripts. Approval in Norway does not permit us to share survey or interview data publicly.
