# Supplementary material for: Identifying Contextual Factors That Shape Cybersecurity Risk Perception for Assisted Living and Health Care Technologies and Wearables: Mixed Methods Study
Source: J Med Internet Res. 2025 Mar 19;27:e64388. doi: 10.2196/64388 (PMC11966077; doi:10.2196/64388)
Supplement: Multimedia Appendix 1 [file jmir_v27i1e64388_app1.pdf]

Survey Questions

Trust and cybersecurity risk perception related to using and sharing of patient-generated health data (PGHD) from wearables

REB# H23-01987

Study summary

This study investigates users' trust and risk perception when using wearable healthcare devices and remote care solutions. We aim to evaluate if there is a difference in the perception of cybersecurity risk between users of wearable healthcare devices that collects patient health data and the people who receive these data. Cybersecurity is *"The protection of computer systems and networks from attack by malicious actors that may result in unauthorized information disclosure, theft of, or damage to hardware, software, or data, as well as from the disruption or misdirection of the services they provide"* [1].

We are recruiting families (parents and/or adolescents), adults, clinicians, researchers using healthcare-related wearable technologies or their data, and IT and medical device professionals who manage or work with such devices. Participants will complete a survey and indicate if they also wish to participate in an optional interview to elaborate on their survey responses and discuss additional relevant scenarios and topics.

Your participation

Your participation in this survey study is voluntary. If you decide to participate, we will ask you to complete this survey, which includes a brief demographics section to tell us a little about yourself, such as your age, and then questions related to the devices you use and the risk you perceive to their data being used and changed/misused by unauthorized parties.

Confidentiality

If you provide your email to be contacted for future interviews, your survey responses will be linked to your email address and so will not be completely anonymous within the project team, i.e., the study administrators will be able to identify you and access your information. If you do not provide your email address, it will be anonymous unless you disclose identifiable information in your free text answers. However, your responses will be de-identified at the close of the study using a unique code, and the project team will remove any information that could easily identify you. De-identified data will be shared with researchers worldwide, published, and used in presentations. The team may also use a quote

[1] Daniel Schatz, Rabih Bashroush, and Julie Wall, 'Towards a More Representative Definition of Cyber Security', *The Journal of Digital Forensics, Security and Law*, 12 (2017), Article 8 <https://doi.org/10.15394/jdfsl.2017.1476>.

from your comments. Summary data from this study will be included in the PhD dissertation of Alvhild Skjelvik, as part of their PhD program in information security and communication technology at the Norwegian University of Science and Technology.

Your confidentiality will be respected. However, research records identifying you may be inspected in the presence of the Investigator or designate and by the University of British Columbia / Children's and Women's Research Ethics Board so they can monitor the research. No information that discloses your identity will be published, removed, or released without your consent unless required by law. All study data will be stored within the BC Children's Hospital Research Institute for five years after any publication of the survey results according to their approved security and privacy standards. Federal and provincial laws legally protect your privacy rights. You also have the right to access information about you and, if need be, correct errors in this information.

You can withdraw from the study anytime, but we cannot remove your de-identified results if you decide to withdraw after your data are analyzed or shared with other researchers.

Participant remuneration

Upon completing the survey, you will be asked to provide your email address if you wish to enter a raffle draw for a $25 electronic gift card (1 gift card for every 10 participants).

This study is funded by a 2020 BC Children's Hospital Research Institute External Salary Recognition Award and further supported by a Michael Smith Health Research BC Scholar Award (M. Görges).

Any questions?

If you have any questions about this study, contact the principal investigator, Dr. Matthias Görges, at mgoerges@bcchr.ubc.ca. If you have any privacy concerns or questions about your rights as a research participant, don't hesitate to get in touch with the Research Participant Compliant Line in the UBC’s Office of Research Ethics by e-mail at RSIL@ors.ubc.ca or by phone at 604-822-8598 (Toll-Free: 1-877-822-8598). Please reference the study number (H23-01987) when contacting the Complaint Line so the staff can better assist you.

By completing the questionnaire, you are consenting to participate in this research. We greatly appreciate your time in our attempt to learn more about the opportunities and risks of patient-generate health data device use and data sharing.

Qualifying questions

1. Which of the following participant groups do you most identify with?
   a) An adolescent (age 15-18), and/or parent of an adolescent, living with type 1 diabetes (T1D) or an adult with T1D who uses continuous glucose monitor and/or insulin pump
   b) An adolescent, or parent of an adolescent, or adult who uses other wellbeing technologies, such as activity trackers
   c) Healthcare provider (HCP)
   d) Healthcare researcher
   e) IT professionals, biomedical engineers or medical device industry professionals familiar with the use of wearable technology in healthcare
   f) None of the above

2. Do you reside in British Columbia?
   - Yes
   - No

[if 1 = not (f) and 2 = Yes, then continue, otherwise display a "Thank you for your interest, but you are not eligible to participate in this study" message and exit]

Demographic questions

3. Your gender is
   - Male
   - Female
   - Agender
   - Two-spirited
   - Transgender
   - Gender Fluid
   - Non-Binary
   - Prefer not to answer
   - Other

4. How old are you?
   - 15 - 20
   - 21 - 25
   - 26 - 30
   - 31 - 35
   - 36 - 40
   - 41 - 45
   - 46 - 50
   - 51 - 55
   - 56 - 60
   - 61 - 65
   - 66 – 70
   - 71-75
   - 76-80
   - Over 80

- Prefer not to answer

5. People living in Canada come from different cultural and racial backgrounds. Please read the following categories and select all that apply to you:
    - Arab
    - Black (e.g., African, Haitian, Jamaican, Somali, etc…)
    - Chinese
    - Filipino
    - Japanese
    - Korean
    - Latin American
    - South Asian (e.g., Pakistani, Sri Lankan, etc…)
    - South East Asian (e.g., Vietnamese, Cambodian, Malaysian, Laotian, etc…)
    - West Asian (e.g., Iranian, Lebanese, Afghan, etc…)
    - Indigenous Canadian
    - White
    - Other
    - Prefer not to answer

6. (If 1 = c, d, or e only) Which of the following best describes your work situation?
    - I work as a clinician in a primary care setting
    - I work as a clinician in specialist healthcare services (such as a hospital)
    - I provide technical or operational support for healthcare systems or devices
    - I perform research using patient-generated health data or data from wearables
    - I don't work in healthcare services
    - I am retired
    - Prefer not to answer
    - Other

    If other, please elaborate on your current situation

7. (If 6 = "I work in a primary care setting" or "I work in specialist healthcare services (such as a hospital" only) only) What is your level of training?
    - Resident physician/ Clinical fellow
    - Nurse/ nurse practitioner
    - Attending Physician
    - Other

    If other, please describe your current level of training ____________

8. (If 6 = "I work in a primary care setting" or "I work in specialist healthcare services (such as a hospital)" or "I provide technical or operational support for healthcare systems or devices" or "I perform research using patient-generated health data or data from wearables" or "Other" only) How many years have you been in practice?
    - 0-5
    - 5-10
    - 10-15

- 15-20
- 20+

9. (If 6 = “I work in a primary care setting” or “I work in specialist healthcare services (such as a hospital” only) In which setting do you typically practice?
   - Academic
   - Community / clinical

10. (If 1 = c, d, or e only) In the past three years have you been involved in any of the following activities:
   - Research
   - Quality assurance/improvement
   - Teaching continuing medical education
   - None of the above

Social media

11. Which of the following social media platforms do you use the most?
    - LinkedIn
    - Facebook
    - Twitter
    - Instagram
    - Snapchat
    - TikTok
    - WeChat
    - WhatsApp
    - Threads
    - Signal
    - Other
    - I don’t use social media

12. Would you be interested in participating in an additional interview (over Zoom) for this research study?
    - Yes
    - No

13. Would you like to be included in the raffle for a $25 Amazon.ca gift card as a remuneration for study involvement?
    - Yes
    - No

14. (If Yes to [12] or [13]) Please enter your email address: _____________

We will use email to communicate about this research study. The research team will use their best efforts to keep your information confidential. However, there are always some risks of disclosure when using email. You should be aware that some email services may store the contents of your email account outside of Canada, where privacy and data security standards may be different than in Canada. If you have questions or would like

to stop receiving research communication via email, don't hesitate to get in touch with the study's principal investigator, Dr Matthias Görges at mgoerges@bcchr.ubc.ca.

Questions for participants working in healthcare only

(i.e. question 6 =“I work in a primary care setting” or “I work in specialist healthcare services (such as a hospital)” or ”I provide technical or operational support for healthcare systems or devices” or “I perform research using patient-generated health data or data from wearables” or “Other”)

15. (If 6 = “I work in a primary care setting” or “I work in specialist healthcare services (such as a hospital)” only) How long have you been working in the healthcare sector?
    - < 1 year
    - 2 – 5 years
    - 6 – 10 years
    - 11 – 15 years
    - 16 – 20 years
    - 21 – 25 years
    - 26 – 30 years
    - 31 - 35 years
    - 36 – 40 years
    - > 40 years

16. How long have you been employed at your current organization?
    - 0 – 5 years
    - 6 – 10 years
    - 11 – 15 years
    - 16 – 20 years
    - 21 – 25 years
    - 26 – 30 years
    - 31 – 35 years
    - 36 – 40 years
    - > 40 years

17. Has your organization or institution ever been a victim of a cyber-attack?
    A cyber-attack is when an attacker (for example, a hacker) tries to or successfully damages or destroys a computer network, system or connected devices or compromise the data stored in computer networks, systems or connected devices. This could involve stealing personal details like credit card numbers or passwords. Many methods can be used, like spreading viruses or tricking people into revealing their passwords for further exploitation.
    In the context of a hospital and healthcare systems, these contain patient data and patient-generated health data (e.g. heart rate, blood pressure, glucose measurements, information from surveys, etc., that the patient/family collets themselves). A cyber-attack in this context could occur if a hacker attempts to break into the system to steal, alter or destroy patient data.
    - Yes
    - No

- I don’t know

18. Do you hold a management position?
    - Yes
    - No

19. Are you using technology as a part of your daily work?
    - Yes
    - No
    - I don’t know

20. Does your organization have an information security policy?
    - Yes
    - No
    - I don’t know

21. (If 20=Yes, then continue) How familiar are you with the content of the security policy?
    - Very unfamiliar
    - Unfamiliar
    - Somewhat familiar
    - Familiar
    - Very Familiar

22. (If 20=Yes, then continue) How would you rate the importance of following the security policy?
    - Not very important
    - Rather important
    - Not sure
    - Important
    - Very important

23. How would you rate your general technical knowledge about computers, mobile devices and the internet?
    - Poor
    - Fair
    - Good
    - Excellent

24. Have you ever received training concerning IT security?
    - Yes
    - No

Common situations

The following questions present everyday situations where you may find yourself while using computers and the internet.

Frequency of your behaviour

25. How often do you lend your e-mail login and password to your friends or relatives?
    - Never
    - Rarely
    - Sometimes
    - Often
    - Always

26. How often do you lend anyone your private debit or credit card(s) and associated PIN(s)?
    - Never
    - Rarely
    - Sometimes
    - Often
    - Always

27. How often do you reveal your PIN (by non-concealment or saying it out loud) when you pay by card?
    - Never
    - Rarely
    - Sometimes
    - Often
    - Always

28. How often do you reveal your e-mail login credentials to others, who are not your friends or relatives?
    - Never
    - Rarely
    - Sometimes
    - Often
    - Always

Ratings of importance

29. How would you rate the importance of updating your smartphone or laptop with the latest software?
    - Not very important
    - Rather important
    - Not sure
    - Important
    - Very important

30. How would you rate the importance of logging off or locking your computer screen when working or studying in public places, if you leave your computer?
    - Not very important
    - Rather important
    - Not sure

- Important
- Very important

31. How would you rate the importance of checking removable media for viruses before usage?
Removable media can be USB-keys, external hard drives, etc.
    - Not very important
    - Rather important
    - Not sure
    - Important
    - Very important

32. How would you rate the importance of regularly changing of your passwords with new ones, at least for frequently used services?
    - Not very important
    - Rather important
    - Not sure
    - Important
    - Very important

33. How would you rate the likelihood of someone stealing your identity on the internet (some examples of services that use your personal identity include online banking, social media, and e-mail)?
    - Very Unlikely
    - Unlikely
    - Neutral
    - Likely
    - Very likely

34. How would you rate the likelihood of someone stealing money from your bank account when using mobile or internet banking?
    - Very Unlikely
    - Unlikely
    - Neutral
    - Likely
    - Very likely

35. How would you rate the likelihood of someone hacking your personal computer, laptop, or smartphone?
    - Very Unlikely
    - Unlikely
    - Neutral
    - Likely
    - Very likely

36. How would you rate the likelihood of losing your private photos and videos?

- Very Unlikely
- Unlikely
- Neutral
- Likely
- Very likely

37. How would you rate the consequence of someone misusing your debit or credit card?
    - Insignificant
    - Minor
    - Moderate
    - Major
    - Catastrophic

38. How would you rate the consequence if someone stole your password to your private accounts?
    - Insignificant
    - Minor
    - Moderate
    - Major
    - Catastrophic

39. How would you rate the consequence if someone stole your password to your work /or study account?
    - Insignificant
    - Minor
    - Moderate
    - Major
    - Catastrophic

40. How would you rate the consequence if someone hacked your computer, laptop, or smartphone?

    - Insignificant
    - Minor
    - Moderate
    - Major
    - Catastrophic

Healthcare specific questions

In this section, we will ask questions related directly to the healthcare sector and the use of technology in healthcare services

41. What types of technology are you familiar with?

When we say "familiar with" it means that you have some knowledge or experience with it. In the context of technology, it means having a general understanding of different types of technology and how they work. It does not necessarily mean that you are an expert or know all the details. But,

instead, you have a basic knowledge and awareness of various technological concepts, devices, or systems.

- Medical device technology (e.g. electrocardiogram, MRI/CT scanner, pacemaker/defibrillator, infusion pumps, insulin pumps, continuous glucose sensors, pacemakers)
- Assisted living technology (e.g. medical alert buttons/system, wearable health monitors, electronic medical dispensers)
- Electronic medical records or electronic personal health records
- Remote care solutions (Video consultations, digital reporting forms, smart scales, smart blood pressure cuffs)
- Wellness devices and mobile applications for health and wellness (e.g. smart watch, exercise tracker, sleep tracking, nutrition diaries, period tracking)
- None of the above

42. (If 41 = any except "None of the above") How did you become familiar with the technology?
    - I am using such technologies as a patient
    - I am using such technologies as a clinician
    - I am familiar with the technologies as a family member of someone using such technology
    - I am familiar with the technologies because I am or have been involved with the management of such technologies
    - I am familiar with the technologies because I am or have been involved with the development of such technologies
    - I know the technology, but have never been directly involved with the use, management or development of such technologies
    - I don't know any such technologies

43. Example 1: Your perception of risk related to Insulin infusion pump/ *continuous glucose monitoring*

An insulin infusion pump is a small, computerized device that helps people with diabetes manage their blood sugar levels. It delivers insulin (a hormone that helps regulate blood sugar) continuously throughout the day through a tiny tube inserted under the skin. Think of it as a mini-computer that you wear which provides a steady flow of insulin, similar to how your body naturally releases insulin.

A continuous glucose monitoring device is a tool used by people with diabetes to keep track of their blood sugar levels in real-time. It consists of a small sensor placed under the skin that measures glucose levels in the tissue fluid. This sensor is connected to a receiver or a smartphone, displaying glucose readings throughout the day. It helps individuals understand how their blood sugar levels change over time, providing valuable information for managing diabetes without needing to prick their fingers for a blood test each time.

For example, if a continuous glucose monitoring device becomes unavailable, the patient will not be able to monitor glucose levels, and the clinician will not receive any data related to glucose monitoring.

There are no right or wrong answers. If you feel you don't know how to answer, please just give us your best guess.

44. Do you use an insulin infusion pump and/or a continuous glucose monitoring device?

- Yes
- No

45. How would you rate the likelihood of the insulin infusion pump/ continuous glucose monitoring being unavailable?
"Unavailability" occurs when the device fails to operate properly or experiences temporary disruptions in delivering accurate and continuous information regarding glucose levels or insulin administration. This can occur due to for example technical malfunctions such as a software glitch, or due to unauthorized access to the device's software or communication channel that may disrupt its functionality which can cause interruptions in providing crucial information generated in the device.
With likelihood, we mean how you perceive the chance that the insulin infusion pump/ glucose monitoring device you are familiar with, becomes unavailable. If unavailable, the technology/device will not function as normal and will be unable to meet its purpose.
    - Very Unlikely
    - Unlikely
    - Neutral
    - Likely
    - Very Likely

46. How would you rate the likelihood of data transmitted from/to the insulin infusion pump/ *glucose monitoring device* being altered?
The alternation of data would in this example happen without your knowledge.
    - Very Unlikely
    - Unlikely
    - Neutral
    - Likely
    - Very Likely

47. How would you rate the likelihood of the data transmitted and stored in the infusion pump/ *glucose monitoring device* being made public?
    - Very Unlikely
    - Unlikely
    - Neutral
    - Likely
    - Very Likely

48. How would you rate the consequence if the infusion pump/ *glucose monitoring device* became unavailable?
    - Insignificant
    - Minor
    - Moderate
    - Major
    - Catastrophic

49. How would you rate the consequence if data transmitted and stored in the infusion pump/ *glucose monitoring device* was made public?
    - Insignificant
    - Minor
    - Moderate
    - Major
    - Catastrophic

50. How would you rate the consequence if data was changed without your knowledge?
    Data is referred to as the data being transmitted and stored by the infusion pump/ *glucose monitoring device. If data is changed, altered or manipulated, this means that the data being transmitted can be changed. For example, if a patient reports glucose levels through the continuous glucose monitoring device, if data was changed unauthorized, wrong values could potentially be shared with the clinician.*
    - Insignificant
    - Minor
    - Moderate
    - Major
    - Catastrophic

Example 2: **Your perception of risk related to smartwatches.**

A smartwatch, resembling a wristwatch, connects to your phone and offers smartphone-like functions such as notifications, calls, and messages. Its touchscreen interface grants easy access to apps and tracks physical activities like steps and heart rate. Some models go further, monitoring sleep patterns, stress levels, and various biomarkers, in addition to having GPS for activity tracking. Essentially, it is a compact extension of your smartphone, providing convenience and comprehensive health-tracking features right on your wrist.

Smartwatches that allow users to collect and share data like step counts, sleep rhythm, motion/activity, location, etc. For example, if a smartwatch becomes unavailable, users cannot monitor their exercise levels and share such data with family and friends.

51. Do you use a smartwatch?
    - Yes
    - No

52. How would you rate the likelihood that the smartwatch was unavailable?
    With the likelihood of unavailability, we mean how you perceive the chance that the smartwatch is not working. If unavailable, the smartwatch/device will not function as expected and will be unable to meet its purpose.
    - Very Unlikely
    - Unlikely
    - Neutral
    - Likely
    - Very Likely

53. How would you rate the likelihood of data transmitted in the smartwatch being altered?
The alternation of data would, in this example, happen without your knowledge.
    - Very Unlikely
    - Unlikely
    - Neutral
    - Likely
    - Very Likely

54. How would you rate the likelihood of the data transmitted and stored in the smart watch being made public?
    - Very Unlikely
    - Unlikely
    - Neutral
    - Likely
    - Very Likely

55. How would you rate the consequence if the smart watch became unavailable?
    - Insignificant
    - Minor
    - Moderate
    - Major
    - Catastrophic

56. How would you rate the consequence if data transmitted and stored in the smart watch was made public?
    - Insignificant
    - Minor
    - Moderate
    - Major
    - Catastrophic

57. How would you rate the consequence if data was changed without your knowledge?
Data are referred to as the data being transmitted and stored by the smartwatch*. If data is changed, altered, or manipulated, this means that the data being transmitted can be changed. For example, if a person monitors their sleep, activity, pulse through a smart watch device, and data were changed by an unauthorized person, incorrect values would be displayed.*
    - Insignificant
    - Minor
    - Moderate
    - Major
    - Catastrophic

Quality of your password

58. What strategy do you use to remember your passwords?
    - Same password for different accounts

- Minor changes in password similarity (e.g reusing word/phrase with added number change)
- Password related to family and birthdays
- Phrases that remind you of the login account
- Password manager
- Write it down somewhere convenient
- Other

59. If we asked you the following question *“For checking the quality of your password security, please write down your most used password”* below – would you provide it to us so that we could check the quality of your password?
    - Yes
    - No
