# Supplementary material for: Identifying Contextual Factors That Shape Cybersecurity Risk Perception for Assisted Living and Health Care Technologies and Wearables: Mixed Methods Study
Source: J Med Internet Res. 2025 Mar 19;27:e64388. doi: 10.2196/64388 (PMC11966077; doi:10.2196/64388)
Supplement: Multimedia Appendix 2 [file jmir_v27i1e64388_app2.pdf]

Nettskjema

Informasjonssikkerhet i helsesektoren - spørreundersøkelse

Hvordan oppfører vi oss på internett?

I denne spørreundersøkelsen vil det bli stilt spørsmål relatert til atferd på internett og bruk av teknologi. Formålet er å bidra med kunnskap for å sikre digitale løsninger på en best mulig måte, hvor perspektivene til ulike grupper som jobber i-, og med helse/teknologi hensyntas.
Denne forskningen er finansiert av Norges teknisk-naturvitenskapelige universitet (NTNU) og godkjent av SIKT (tidligere Norsk Senter for Forskningsdata (NSD)). Alle svar er **anonyme** og **konfidensielle**. Innsamlede data vil bli lagret kryptert og vil slettes etter analyse.

Tusen takk for at du deltar!
Ved spørsmål eller andre henvendelser kan du kontakte Alvhild Skjelvik (alvhild.skjelvik@ntnu.no)

Bakgrunnsinformasjon

Ditt kjønn er

Mann
Kvinne
Ønsker ikke å oppgi

Hvor gammel er du?

20 - 25 år
26 - 30 år
31 - 35 år
36 - 40 år
41 - 45 år
46 - 50 år
51 - 55 år
56 - 60 år
61 - 65 år
66 - 70 år
71-75 år
76-80 år
Over 80 år

Hvilken beskrivelse passer best til din arbeidssituasjon?

Jeg jobber i primærhelsetjenesten (kommune, fastlege, hjemmesykepleien)
Jeg jobber i spesialisthelsetjenesten (sykehus, regionalt helseforetak)
Jeg jobber i privat sektor (privat sykehus, helseorganisasjon, IT-organisasjon)
Jeg besvarer denne spørreundersøkelsen som pasient
Annet

Hvor lenge har du jobbet i/med helsesektoren?

This element is only shown when the option 'Jeg jobber i primærhelsetjenesten (kommune, fastlege, hjemmesykepleien) or Jeg jobber i spesialisthelsetjenesten (sykehus, regionalt helseforetak) or Jeg jobber i privat sektor (privat sykehus, helseorganisasjon, IT-organisasjon) or Annet' is selected in the question 'Hvilken beskrivelse passer best til din arbeidssituasjon?'

Under 1 år

2 - 5 år

6 - 10 år

11 - 15 år

16 - 20 år

21 - 25 år

26 - 30 år

Over 30 år

Jeg jobber ikke i helsesektoren

Hvor lenge har du vært ansatt i denne virksomheten?

This element is only shown when the option 'Under 1 år or 2 - 5 år or 6 - 10 år or 11 - 15 år or 16 - 20 år or 21 - 25 år or 26 - 30 år or Over 30 år or Jeg jobber ikke i helsesektoren' is selected in the question 'Hvor lenge har du jobbet i/med helsesektoren?'

0 - 5 år

6 - 10 år

11 - 15 år

16 - 20 år

21 - 25 år

26 - 30 år

Over 30 år

Har virksomheten din blitt utsatt for et dataangrep?

This element is only shown when the option '0 - 5 år or 6 - 10 år or 11 - 15 år or 16 - 20 år or 21 - 25 år or 26 - 30 år or Over 30 år' is selected in the question 'Hvor lenge har du vært ansatt i denne virksomheten?'

Ja

Nei

Jeg vet ikke

Har du en lederstilling?

This element is only shown when the option 'Ja or Nei or Jeg vet ikke' is selected in the question 'Har virksomheten din blitt utsatt for et dataangrep?'

(Personer som rapporterer til deg)

Ja

Nei

Bruker du informasjonsteknologi som en del av ditt daglige arbeid?

This element is only shown when the option 'Ja or Nei or Jeg vet ikke' is selected in the question 'Har virksomheten din blitt utsatt for et dataangrep?'

Ja

Nei

Jeg vet ikke

Hva beskriver best din nåværende stilling?

This element is only shown when the option 'Under 1 år or 2 - 5 år or 6 - 10 år or 11 - 15 år or 16 - 20 år or 21 - 25 år or 26 - 30 år or Over 30 år' is selected in the question 'Hvor lenge har du jobbet i/med helsesektoren?'

Ledelse/ administrasjon

Hjelpepleier, vernepleier eller helsefagarbeider

Sykepleier

Lege/fastlege

IT-personell/ IT-spesialist

Teknologileverandør

Annet

Hvis du valgte teknologileverandør - hvilken av de følgende alternative passer best for å beskrive din stilling

This element is only shown when the option 'Teknologileverandør ' is selected in the question 'Hva beskriver best din nåværende stilling?'

Ledelse/administrasjon

IT/ software/ utvikling

Sikkerhet

Operations/ drift

Juridisk

HR

Økonomi

Kommunikasjon

Hvis du valgte IT-personell / IT-spesialist, hvilken av de følgende alternative passer best for å beskrive din stilling

This element is only shown when the option 'IT-personell/ IT-spesialist' is selected in the question 'Hva beskriver best din nåværende stilling?'

Jeg jobber i en privat IT-organisasjon

Jeg jobber i en offentlig IT-organisasjon

Jeg jobber hos en leverandør av teknologi

Har organisasjonen din en informasjonssikkerhetspolicy?

This element is only shown when the option 'Jeg jobber i primærhelsetjenesten (kommune, fastlege, hjemmesykepleien) or Jeg jobber i spesialisthelsetjenesten (sykehus, regionalt helseforetak) or Jeg jobber i privat sektor (privat sykehus, helseorganisasjon, IT-organisasjon) or Annet' is selected in the question 'Hvilken beskrivelse passer best til din arbeidssituasjon?'

Ja

Nei

Jeg vet ikke

Hvor kjent er du med innholdet i sikkerhetspolicyen?

This element is only shown when the option 'Ja' is selected in the question 'Har organisasjonen din en informasjonssikkerhetspolicy?'

Svært ukjent

Ukjent

Noe kjent

Kjent

Svært kjent

Hvordan vurderer du viktigheten av å følge sikkerhetspolicyen?

This element is only shown when the option 'Ja' is selected in the question 'Har organisasjonen din en informasjonssikkerhetspolicy?'

Ikke så viktig

Ganske viktig

Usikker

Viktig

Svært viktig

Hvordan vil du vurdere din generelle tekniske kunnskap om datamaskiner og internett?

Dårlig

Middels

God

Utmerket

Har du noensinne fått opplæring i IT-sikkerhet?

E-læring eller personlig opplæring med temaer som sosial manipulasjon, løsepengevirus, phishing/nettfisking, organisasjonens sikkerhetspolicy.

Ja

Nei

Vanlige situasjoner

Følgende spørsmål omhandler hverdagssituasjoner som du kan befinne deg når du bruker datamaskiner og internett.

Hyppigheten av din oppførsel

Hvor ofte låner du ut e-post pålogging og passord til venner eller slektninger?

Aldri

Sjeldent (årlig)

Noen ganger (månedlig)

Ofte (ukentlig)

Alltid (daglig)

Hvor ofte låner du ut ditt bank- eller kredittkort og tilhørende PIN-koder til noen?

Aldri

Sjeldent (årlig)

Noen ganger (månedlig)

Ofte (ukentlig)

Alltid (daglig)

Hvor ofte avslører du PIN-koden (ved å ikke skjule den eller si den høyt) når du betaler med kort?

Aldri

Sjeldent (årlig)

Noen ganger (månedlig)

Ofte (ukentlig)

Alltid (daglig)

Hvor ofte avslører du påloggingsinformasjonen som passord/ brukernavn til personlige kontoer til andre?

Andre kan være kollegaer, bekjente og lignende.

- Aldri
- Sjeldent (årlig)
- Noen ganger (månedlig)
- Ofte (ukentlig)
- Alltid (daglig)

Viktigheten av din oppførsel

Hvordan vil du vurdere viktigheten av å oppdatere din smarttelefon eller bærbare datamaskin med siste programvare?

Med siste programvare menes siste versjon av en programvare, regelmessige oppdateringer og liknende.

- Ikke så viktig
- Ganske viktig
- Usikker
- Viktig
- Svært viktig

Hvordan vil du vurdere viktigheten av å logge av eller låse din dataskjerm og/eller smarttelefon når du arbeider på offentlige steder?

- Ikke så viktig
- Ganske viktig
- Usikker
- Viktig
- Svært viktig

Hvordan vil du vurdere viktigheten av å sjekke flyttbare medier (enheter som kan kobles til datamaskinen) for virus før du bruker dem?

USB-minnepinner, CD, harddisker, osv.

- Ikke så viktig
- Ganske viktig
- Usikker
- Viktig
- Svært viktig

Hvordan vil du vurdere viktigheten av å regelmessig endre og oppdatere dine passord, i det minste for tjenester du bruker ofte?

- Ikke så viktig
- Ganske viktig
- Usikker
- Viktig
- Svært viktig

Sosiale medier

Hvilke av de følgende sosiale mediene bruker du mest?

LinkedIn
Facebook
Twitter
Instagram
Snapchat
TikTok
WeChat
Whatsapp
Jeg bruker ikke sosiale medier
Andre

Hvordan vil du vurdere sannsynligheten for at noen stjeler identiteten din på internett ?

Eksempelvis på din nettbank, Facebook, din e-post.

Svært usannsynlig
Usannsynlig
Nøytral
Sannsynlig
Svært sannsynlig

Hvordan vil du vurdere sannsynligheten for at noen stjeler penger fra bankkontoen din når du bruker mobil- eller nettbank?

Svært usannsynlig
Usannsynlig
Nøytral
Sannsynlig
Svært sannsynlig

Hvordan vil du vurdere sannsynligheten for at noen hacker din personlige datamaskin, bærbare PC eller smarttelefon?

Svært usannsynlig
Usannsynlig
Nøytral
Sannsynlig
Svært sannsynlig

Hvordan vil du vurdere sannsynligheten for å miste private bilder og videoer?

Svært usannsynlig
Usannsynlig
Nøytral
Sannsynlig
Svært sannsynlig

Hvordan vil du vurdere sannsynligheten for at noen misbruker bank- eller

kredittkortet ditt?

Svært usannsynlig
Usannsynlig
Nøytral
Sannsynlig
Svært sannsynlig

Hvordan vil du vurdere konsekvensen hvis noen stjeler passordet til dine private kontoer?

Ubetydelig
Liten
Moderat
Stor
Katastrofal

Hvordan vil du vurdere konsekvensen hvis noen stjeler passordet til din arbeidskonto?

This element is only shown when the option ‘Jeg jobber i primærhelsetjenesten (kommune, fastlege, hjemmesykepleien) or Jeg jobber i spesialisthelsetjenesten (sykehus, regionalt helseforetak) or Jeg jobber i privat sektor (privat sykehus, helseorganisasjon, IT-organisasjon) or Annet’ is selected in the question ‘Hvilken beskrivelse passer best til din arbeidssituasjon?’

Ubetydelig
Liten
Moderat
Stor
Katastrofal

Hvordan vil du vurdere konsekvensen hvis noen hacker din personlige datamaskin, bærbare PC eller smarttelefon?

Ubetydelig
Liten
Moderat
Stor
Katastrofal

Spesifikke spørsmål knyttet til helsesektoren og helsetjenester

I denne delen vil vi stille spørsmål relatert direkte til helsetjenesten og bruk av teknologi i helsetjenesten.

Hvilke(n) type(r) teknologi er du kjent med?

Når vi sier "kjent med" - betyr det at du har noe kunnskap eller erfaring med den, og at du er involvert i bruk, drift, forvaltning og/eller implementering av den. I konteksten av teknologi betyr det å ha en generell forståelse av forskjellige typer teknologi og hvordan de fungerer. Det betyr ikke nødvendigvis at du er ekspert eller kjenner alle detaljer, men heller å ha en grunnleggende kunnskap og bevissthet om ulike teknologiske konsepter, enheter eller systemer.

Medisinsk utstyr (EKG, MR/CT, hjertedefibrillator, infusjonspumper, pacemakere)
Velferdsteknologi (Digital trygghetsalarm, elektronisk medisindispenser, lokaliseringsteknologi,

robotstøvsuger, etc.)
Digital pasientjournal/ elektronisk pasientjournal
Digital hjemmeoppfølging (video-/telefonsamtaler med helsepersonell, digitale skjema.)
Mobilapplikasjoner for helse og velvære (trening, kosthold, søvnsporing, osv.)
Virtuell virkelighet i helsetjenesten
Bruk av kunstig intelligens i helsetjenesten
Jeg er ikke kjent med noen slike teknologier
Annet

Hvis du svarte "annet" på forrige spørsmål, vennligst spesifiser hvilken teknologi du er kjent med

This element is only shown when the option ‘Annet’ is selected in the question ‘Hvilke(n) type(r) teknologi er du kjent med?’

Hvordan kjenner du til teknologien?

Vi stiller dette spørsmålet for å forstå din kunnskap og kjennskap til teknologien

Jeg bruker slik teknologi som pasient
Jeg bruker slik teknologi som helsepersonell
Jeg er kjent med teknologien som pårørende til noen som bruker slik teknologi
Jeg er kjent med teknologien fordi jeg er eller har vært involvert i forvaltningen av slike teknologier
Jeg er kjent med teknologien fordi jeg er eller har vært involvert i utviklingen av slike teknologier
Jeg kjenner teknologien, men har aldri vært direkte involvert i bruken, forvaltningen eller utviklingen av slike teknologier
Jeg kjenner ikke til noen slike teknologier.

Din forståelse for bruk av ulik type teknologi - eksempel 1

I de følgende spørsmål tar vi utgangspunkt i bruken av spesifikk teknologi, nærmere bestemt bruk av digitale medisindispensere **eller** bruk av digital selvrapportering. Du bes velge hvilken i neste spørsmål. Vi ber deg om å gjøre en vurdering, basert på din kunnskap og forståelse, i spørsmål relatert til teknologien. Det finnes ingen riktige eller gale svar på disse spørsmålene - vi søker innsikt i din forståelse.

Velg en av de følgende

This element is only shown when the option ‘Jeg bruker slik teknologi som pasient or Jeg bruker slik teknologi som helsepersonell or Jeg er kjent med teknologien som pårørende til noen som bruker slik teknologi or Jeg er kjent med teknologien fordi jeg er eller har vært involvert i forvaltningen av slike teknologier or Jeg er kjent med teknologien fordi jeg er eller har vært involvert i utviklingen av slike teknologier or Jeg kjenner teknologien, men har aldri vært direkte involvert i bruken, forvaltningen eller utviklingen av slike teknologier’ is selected in the question ‘Hvordan kjenner du til teknologien?’

Beskrivelse:
Medisindispensere (digital medisindispenser) er en teknologi som benyttes for å gi medisin, hvor forhåndsdefinerte doser gis til gitte tidspunkt. Dette kan være en eller flere ganger om dagen, og det vil vanligvis gis en beskjed eller et signal fra maskinen når medisinen skal tas.
Digital selvrapportering vil ofte skje via et skjema på et digitalt verktøy (feks iPad, mobilapplikasjon), hvor man deler opplysninger knyttet til sin egen helsetilstand. Det kan innebære hvordan man føler seg, livskvalitet, om man opplever smerter, ubehag og/eller endringer i symptomer knyttet til sin helsetilstand og bruk av medikameneter. Disse opplysningene deles med helsepersonell slik at de kan følge opp pasienter på en god måte, uten at pasienten selv må dra til sykehus/lege eller motta besøk av helsepersonell.

Medisindispenser
Digital selvrapportering

Hvordan vil du vurdere sannsynligheten for at medisindispenser ikke er tilgjengelig?

This element is only shown when the option 'Medisindispenser' is selected in the question 'Velg en av de følgende'

Dersom medisindispenser ikke er tilgjengelig vil den ikke gjøre den/de oppgavene teknologien er ment til å gjøre, og den vil være helt eller delvis ut av stand til å fungere som normalt.

- Svært usannsynlig
- Usannsynlig
- Nøytral
- Sannsynlig
- Svært sannsynlig

Hvordan vil du vurdere sannsynligheten for at digitalt selvrapporteringsskjema ikke er tilgjengelig?

This element is only shown when the option 'Digital selvrapportering' is selected in the question 'Velg en av de følgende'

Dersom selvrapporteringsskjema ikke er tilgjengelig vil den ikke gjøre den/de oppgavene teknologien er ment til å gjøre, og den vil være helt eller delvis ut av stand til å fungere som normalt.

- Svært usannsynlig
- Usannsynlig
- Nøytral
- Sannsynlig
- Svært sannsynlig

Hvordan vil du vurdere sannsynligheten for at data som overføres i medisindispenser blir endret?

This element is only shown when the option 'Medisindispenser' is selected in the question 'Velg en av de følgende'

Endringen av dataene vil i dette tilfellet skje uten din kunnskap om endringen. Med data mener vi data som blir overført og lagret medisindispenseren.

- Svært usannsynlig
- Usannsynlig
- Nøytral
- Sannsynlig
- Svært sannsynlig

Hvordan vil du vurdere sannsynligheten for at data som overføres i selvrapporteringsskjemaet blir endret?

This element is only shown when the option 'Digital selvrapportering' is selected in the question 'Velg en av de følgende'

Endringen av dataene vil i dette tilfellet skje uten din kunnskap om endringen. Med data mener vi data som blir overført og lagret selvrapporteringsskjemaet.

- Svært usannsynlig
- Usannsynlig
- Nøytral
- Sannsynlig
- Svært sannsynlig

Hvordan vil du vurdere sannsynligheten for at dataene som blir overført og lagret i en medisindispenser blir gjort offentlig tilgjengelige?

This element is only shown when the option 'Medisindispenser' is selected in the question 'Velg en av de følgende'

Med data mener vi data som blir overført og lagret i medisindispenser.

- Svært usannsynlig
- Usannsynlig
- Nøytral
- Sannsynlig
- Svært sannsynlig

Hvordan vil du vurdere sannsynligheten for at dataene som blir overført og lagret i selvrapporteringsskjema blir gjort offentlig tilgjengelige?

This element is only shown when the option 'Digital selvrapportering' is selected in the question 'Velg en av de følgende'

Med data mener vi data som blir overført og lagret i selvrapporteringsskjema.

- Svært usannsynlig
- Usannsynlig
- Nøytral
- Sannsynlig
- Svært sannsynlig

Hvordan vil du vurdere konsekvensen dersom medisindispenseren ikke var tilgjengelig?

This element is only shown when the option 'Medisindispenser' is selected in the question 'Velg en av de følgende'

- Ubetydelig
- Liten
- Moderat
- Stor
- Katastrofal

Hvordan vil du vurdere konsekvensen dersom selvrapporteringsskjemaet ikke var tilgjengelig?

This element is only shown when the option 'Digital selvrapportering' is selected in the question 'Velg en av de følgende'

- Ubetydelig
- Liten
- Moderat
- Stor
- Katastrofal

Hvordan vil du vurdere konsekvensen dersom dataene som blir overført og lagret i medisindispenseren blir gjort offentlig tilgjengelige?

This element is only shown when the option 'Medisindispenser' is selected in the question 'Velg en av de følgende'

Med data mener vi data som blir overført og lagret i medisindispenser.

- Ubetydelig
- Liten
- Moderat
- Stor
- Katastrofal

Hvordan vil du vurdere konsekvensen dersom dataene som blir overført og lagret i

selvrapporteringsskjemaet blir gjort offentlig tilgjengelige?

This element is only shown when the option ‘Digital selvrapportering’ is selected in the question ‘Velg en av de følgende’

Med data mener vi data som blir overført og lagret i selvrapporteringsskjema.

- Ubetydelig
- Liten
- Moderat
- Stor
- Katastrofal

Hvordan vil du vurdere konsekvensen dersom data ble endret uten din kunnskap?

This element is only shown when the option ‘Medisindispenser’ is selected in the question ‘Velg en av de følgende’

Med data mener vi data som blir overført og lagret i medisindispenser.

- Ubetydelig
- Liten
- Moderat
- Stor
- Katastrofal

Hvordan vil du vurdere konsekvensen dersom data ble endret uten din kunnskap?

This element is only shown when the option ‘Digital selvrapportering’ is selected in the question ‘Velg en av de følgende’

Med data mener vi data som blir overført og lagret i selvrapporteringsskjema.

- Ubetydelig
- Liten
- Moderat
- Stor
- Katastrofal

Din forståelse for bruk av ulik type teknologi - eksempel 2

I de følgende spørsmål tar vi utgangspunkt i bruken av spesifikk teknologi, nærmere bestemt bruk av smart klokke. Vi ber deg om å gjøre en vurdering, basert på din kunnskap og forståelse, i spørsmål relatert til smart klokker spesifkt. Det finnes ingen riktige eller gale svar på disse spørsmålene - vi søker innsikt i din forståelse.
En smart klokke har mer funksjonalitet enn tradisjonelle klokker gjennom å ha ulike programvarer installert, og vil ofte ha muligheten til å kobles opp til mobiltelefoner. Smart klokker inneholder ofte sensorer som pulsmåler, skritteller og søvnmonitorering for å nevne noen eksempler, og kan dele denne informasjonen med applikasjoner på mobiltelefoner/ og gjennom ulike applikasjoner.

Hvordan vil du vurdere sannsynligheten for at smart klokken ikke er tilgjengelig?

This element is only shown when the option ‘Jeg bruker slik teknologi som pasient or Jeg bruker slik teknologi som helsepersonell or Jeg er kjent med teknologien som pårørende til noen som bruker slik teknologi or Jeg er kjent med teknologien fordi jeg er eller har vært involvert i forvaltningen av slike teknologier or Jeg er kjent med teknologien fordi jeg er eller har vært involvert i utviklingen av slike teknologier or Jeg kjenner teknologien, men har aldri vært direkte involvert i bruken, forvaltningen eller utviklingen av slike teknologier’ is selected in the question ‘Hvordan kjenner du til teknologien?’

Dersom smart klokken ikke er tilgjengelig vil den ikke gjøre den/de oppgavene smart klokken er ment til å gjøre, og den vil være helt eller delvis ut av stand til å fungere som normalt.

- Svært usannsynlig
- Usannsynlig
- Nøytral
- Sannsynlig

Svært sannsynlig

Hvordan vil du vurdere sannsynligheten for at data som overføres i smart klokken blir endret?

This element is only shown when the option 'Svært usannsynlig or Usannsynlig or Nøytral or Sannsynlig or Svært sannsynlig' is selected in the question 'Hvordan vil du vurdere sannsynligheten for at medisindispenser ikke er tilgjengelig?'

Endringen av dataene vil i dette tilfellet skje uten din kunnskap om endringen. Med data mener vi data som blir overført og lagret i smart klokken.

Svært usannsynlig

Usannsynlig

Nøytral

Sannsynlig

Svært sannsynlig

Hvordan vil du vurdere sannsynligheten for at dataene som blir overført og lagret i smart klokken blir gjort offentlig tilgjengelige?

This element is only shown when the option 'Svært usannsynlig or Usannsynlig or Nøytral or Sannsynlig or Svært sannsynlig' is selected in the question 'Hvordan vil du vurdere sannsynligheten for at data som overføres i medisindispenser blir endret?'

Med data mener vi data som blir overført og lagret i smart klokken.

Svært usannsynlig

Usannsynlig

Nøytral

Sannsynlig

Svært sannsynlig

Hvordan vil du vurdere konsekvensen dersom dataene som blir overført og lagret i smart klokken blir gjort offentlig tilgjengelige?

This element is only shown when the option 'Ubetydelig or Liten or Moderat or Stor or Katastrofal' is selected in the question 'Hvordan vil du vurdere konsekvensen dersom medisindispenseren ikke var tilgjengelig?'

Med data mener vi data som blir overført og lagret i smart klokken.

Ubetydelig

Liten

Moderat

Stor

Katastrofal

Hvordan vil du vurdere konsekvensen dersom smart klokken ikke var tilgjengelig?

This element is only shown when the option 'Svært usannsynlig or Usannsynlig or Nøytral or Sannsynlig or Svært sannsynlig' is selected in the question 'Hvordan vil du vurdere sannsynligheten for at dataene som blir overført og lagret i en medisindispenser blir gjort offentlig tilgjengelige?'

Ubetydelig

Liten

Moderat

Stor

Katastrofal

Hvordan vil du vurdere konsekvensen dersom dataene i smart klokken ble endret uten din kunnskap?

This element is only shown when the option 'Ubetydelig or Liten or Moderat or Stor or Katastrofal' is selected in the question 'Hvordan vil du vurdere konsekvensen dersom dataene som blir overført og lagret i medisindispenseren blir gjort offentlig tilgjengelige?'

Med data mener vi data som blir overført og lagret i smart klokken.

- Ubetydelig
- Liten
- Moderat
- Stor
- Katastrofal

Kvaliteten på ditt passord

Hvilken strategi bruker du for å huske passordene dine?

- Samme passord for ulike kontoer
- Mindre endringer i passordlikhet (for eksempel gjentakelse av ord/frase med tillegg av tallendringer)
- Passord relatert til familie og bursdager
- Setninger som minner deg om påloggingskontoen
- Passordlagringsverktøy
- Annet

For å sjekke kvaliteten på passordet ditt, skriv ned ditt mest brukte passord:
