# Supplementary material for: Identifying Contextual Factors That Shape Cybersecurity Risk Perception for Assisted Living and Health Care Technologies and Wearables: Mixed Methods Study
Source: J Med Internet Res. 2025 Mar 19;27:e64388. doi: 10.2196/64388 (PMC11966077; doi:10.2196/64388)
Supplement: Multimedia Appendix 3 [file jmir_v27i1e64388_app3.pdf]

Interview guide

Note: Adjust questions and language of questions (wording, terminology, complexity) based on the respondent being interviewed.

Thank you for taking the time to sit down with us. We appreciate your participation in this study. My name is [*facilitator name*], and joining us is/are my fellow researcher(s) [*notetaker name(s) if present*]. We are team members working with Dr. Matthias Görges at BC Children's Hospital and the University of British Columbia.

Our study investigates users' trust and risk perception when using wearables and remote care solutions. We aim to evaluate if there is a difference in the perception of cybersecurity risk amongst users of wearable medical devices and other generators of patient-generated health data, such as smartwatches, and the data's recipients, such as doctors.

We define cybersecurity as *"The protection of computer systems and networks from attack by malicious actors that may result in unauthorized information disclosure, theft of, or damage to hardware, software, or data, as well as from the disruption or misdirection of the services they provide"*.

The interview will last roughly forty-five to sixty minutes and will be recorded for further analysis. This interview is confidential, and your name and contact information will be kept separate from the other information you provide in this interview. Recordings and notes of your interview will be kept in a secure database, and only the research team will have access.

You can skip any questions you prefer not to answer and end the interview anytime. You can withdraw from the study even after completing the interview by asking us to remove your interview and information from the database. Do you have any questions about the interview?

Before we start, please confirm your consent to participate and be recorded.
[If consent is not confirmed, thank the participant for their time and end the session.]
[Proceed if consent is confirmed]

Introduction and background

Note to interviewer: *This set of questions is a guide only. It will not be possible to ask them all. Questions selected from each question will be guided by context, previous answers, and responses to the survey. Please ensure that at least one question from each section is asked. The most relevant questions for exploring risk perception are highlighted in green text.*

You completed an online survey before coming to this interview, and this interview will explore some of these topics in more detail. We only want you to answer questions you are comfortable with, and there is no right or wrong answer for some of the questions.

- Can you introduce yourself and briefly describe your role in the [participant category]?

Technology adoption and use

- Which healthcare technologies have you personally adopted and integrated into your daily routine? Simplified version: Which healthcare devices do you use daily?
- How have these healthcare technologies impacted your daily life and routines? Do you find them convenient or beneficial? Simplified version: How do these healthcare devices affect your everyday life? Do you like using them because they are helpful, handy, and accessible?
- Are there any specific healthcare technologies you've hesitated to adopt or use? If so, what concerns or reservations do you have about them? Simplified version: Are there any devices you've been unsure about using? If so, what has made you worry about it?
- Besides convenience and perceived benefits, are any additional factors influencing your decision to adopt and use healthcare technologies? Simplified version: Are there any specific reasons that would make you want to use healthcare devices?

Information security and policies

- Have you ever considered data confidentiality, integrity, and availability when using wearable devices and remote healthcare technology? Can you provide examples or scenarios that come to mind? Simplified version: Have you considered how data is kept secret, if it is correct/true and if you can see your data when using devices?
- How concerned are you about your health and personal information privacy in the context of wearable devices and remote care technology? What are specific privacy-related aspects that worry you? Simplified version: How worried are you about your data when using these devices? Is anything specific that worries you?
- Have you ever experienced situations where you felt that your health data or personal information might be at risk while using healthcare technology? Please provide examples. Simplified version: Have you ever been in situations where you thought your health data or personal data could be in danger when using devices/healthcare technology?
- How confident do you recognize potential security threats or breaches when using healthcare technology? Simplified version: Are you sure you can tell when something is not right with the security of your device?

Risk perception

- Can you share your understanding of cybersecurity risks in the context of the devices we discussed? Simplified version: What do you think are the cybersecurity risks of using the device we talked about?

- Do you feel that the benefits of using devices outweigh the potential risks? How do you weigh these factors in your decision-making? Simplified version: Do you think the benefits of using the devices are worth the possible problems? How would you decide?
- What kind of data is stored within the technology you use? What health and personal information is involved? Simplified version: What information is saved on the devices? What kind of health and personal data?
- How do you perceive the overall risk related to the healthcare technology and digital home monitoring you use? Simplified version: How safe or risky do you think the healthcare technology and home monitors are?
- What is the worst-case scenario you can imagine regarding the technology you use? Simplified version: What is the worst thing that can happen to the technology you use?

Specific technology scenarios

- High-risk device, e.g. continuous glucose monitor
    - Please describe how you use such a device in your daily life.
    - Do you trust the device?
    - What are your expectations of responsibility for the security of this device?
- Low-risk device, e.g. smartwatch
    - Please describe how you use such a device in your daily life.
    - Do you trust the device?
    - What are your expectations of responsibility for the security of this device?

Protection motivation theory

- To what extent do you feel capable of effectively dealing with cybersecurity incidents involving your healthcare technology? Simplified version: Do you think you can handle cybersecurity issues affecting your device well?
- How familiar are you with the security features and practices recommended for your healthcare technology? Simplified version: Do you know much about the security actions you should do with your device?
- How motivated are you to follow security recommendations and practice safety measures with your healthcare technology? Simplified version: How much do you want to follow security rules to ensure your device is secure?
- How confident are you in identifying and responding to cybersecurity incidents that might affect your healthcare technology? Simplified version: Could you tell if something negative/bad related to cybersecurity happened to your device and what to do?
- Do you believe engaging in secure behaviour when using healthcare technology significantly reduces the risks? Simplified version: Do you think your behaviour (you behaving securely) related to your device makes a difference in reducing cybersecurity risks?

Everyday behaviours and cybersecurity

- How often do you think about your personal devices and data security? Simplified version: How often do you worry about the security of your device and data?
- How much trust do you place in the organizations or entities that provide healthcare technology to safeguard your data? Simplified version: Do you trust the companies/organizations providing devices to keep your data safe?

- Can you share any concerns or reservations about the privacy of your health-related data when using technology? Simplified version: Do you worry about your health data's privacy when you use devices/technology?
- Are there specific situations or contexts where you become more cautious about your healthcare data privacy? Simplified version: Are there times when you are more careful about keeping your healthcare data private?

Conclusion
- Are there any topics or concepts you missed or expected us to discuss?
- Do you have anything else to add?

Thank the participant for their time and verify that they wish to receive the $25 gift card for their participation by email.
