# Supplementary material for: Identifying Contextual Factors That Shape Cybersecurity Risk Perception for Assisted Living and Health Care Technologies and Wearables: Mixed Methods Study
Source: J Med Internet Res. 2025 Mar 19;27:e64388. doi: 10.2196/64388 (PMC11966077; doi:10.2196/64388)
Supplement: Multimedia Appendix 4 [file jmir_v27i1e64388_app4.pdf]

Skjelvik et al. (2024). Identifying contextual factors that shape cybersecurity risk perception for assisted living technologies and wearables – a mixed-methods study

Intervju guide (leverandør)

Dato:	
Navn:	
Rolle:	

Intervjuguide

- Introduksjon
    - Kan du fortelle litt om deg selv og din rolle i selskapet?
    - Hvor lenge har du jobbet innenfor området velferdsteknologi?

- Teknologi
    - Kan du fortelle meg litt om den teknologien dere leverer?
    - Hvilket løsninger og konkret utstyr leverer dere?
    - Hvordan kobles utstyret opp? nettverksoppkobling
      Integrerer sensorer i løsningen –
      Laste ned app på hvilken som helst device?

- Utviklingsprosess
    - Hvordan jobber dere med utvikling av tjeneste/løsning?
    - Følges en spesifikk arbeidsmetode – e.g smidig?
    - Hvordan hensyntas sikkerhet i utviklingsprosessen?
    - Hvilken del av utstyrets livssyklus er dere involvert i?
    - Gjennomfører dere sikkerhetstester av deres utstyr mht. Cybersikkerhet?

    - Underleverandører
        - Benytter dere underleverandører for å levere deres løsninger?
          Hvis JA
        - *Hva leverer deres underleverandører i løsningene?*
        - *Hvordan er ansvaret for sikkerhet distribuert?*
        - *Hvordan er databehandler/ansvarlig fordelt?*
        - *Hvordan samhandler dere?*

- Samhandling og samarbeid
    - Hvordan samarbeider dere med kunder om sikkerhet?
        - På hvilke områder jobber dere sammen?
    - Hvem har ansvar for sikkerhet i løsningene når kunden tar dem i bruk?
    - Hvilke forventninger har kundene til dere innenfor sikkerhet?
    - Har dere delt opp deres infrastruktur og nettverk mellom kunder?
    - Har det blitt gjennomført sikkerhetsrevisjoner eller sikkerhetsgjennomganger av deres løsninger?

- Cybersikkerhet/ sikkerhet
    - Hva er ditt forhold til cybersikkerhet?
    - Hvordan jobber dere med sikkerhet i selskapet?
    - Opplever du at sikkerhet er noe dere tar på alvor?
    - Hvem er ansvarlig for sikkerhet i deres løsning/tjeneste?
    - Opplever dere at deres løsning/tjenester er eller kan bli utsatt for uønskede hendelser knyttet til cybersikkerhet?
        - Er det realistisk at løsningene vil bli angrepet?

- Hvor alvorlig opplever dere at cybersikkerhetshendelser mot deres løsning kan bli?
    - Hvilke konsekvenser kan dette medføre?
- Har skalering av deres løsninger effekt på cybersikkerhet?
    - Hvis ja, hvordan har skalering effekt på cybersikkerhet?
- I hvilken grad opplever dere at selskapet er i stand til å motstå cybersikkerhetshendelser rettet mot deres løsning/tjeneste?
- Har dere implementert sikkerhetstiltak som dere pålegger deres kunder?
    - F.eks. relatert til tilgang, passord, standard kryptering, nettverkssementering.

- Innenfor sikkerhetsarbeidet snakker man i enkelte rammeverk om ulike faser for sikkerhetsarbeidet
    - **Evne til å kjenne helheten i løsningene**, alle avhengigheter, verdikjedene i løsningen fra sensorer til varsling, og sårbarheter knyttet til disse?
    - **Evne til å beskytte**, sørge for at de ulike dele av løsningen er oppdatert, konfigurert på en sikker måte
        - Har dere sikkerhetsverktøy, personell med riktig kompetanse som dekker verdikjeden i løsningen (oppdage nedetid, at deler av løsningen ikke fungerer, oppdage og lukke sårbarheter, avdekke uønsket aktivitet)?
    - **Evne til å oppdage** om sikkerhetshendelser skulle inntreffe i ulike deler av løsningen? Også ute i hjemmet, eks om noen "utforsker" løsningen, angriper sentrale komponenter?
    - **Evne til å respondere** og håndtere om en sikkerhetshendelse skulle inntreffe
- Opplever dere at det finnes gode ressurser, rammeverk, lover/reguleringer på sikkerhetsområde som styrker cybersikkerhet og eventuelt reduserer risiko innenfor området?

- Risiko
    - **Kan du fortelle meg litt om din forståelse for risiko relatert til cybersikkerhet?**
    - Hvilke trusler er relevante for deres løsning/tjenester/utstyr?
    - Hvilke sårbarheter kan dere bli eksponert for?
        - Hvordan håndterer dere sårbarheter som blir identifisert?
    - Hvilke data lagres i deres løsning?
        - Hvilke type helse- og personopplysninger?
        - Hvilke tilganger har dere til systemene?
    - Hvordan opplever dere risikoen knyttet til deres løsning/tjeneste?
    - Hvordan arbeider dere for å redusere denne risikoen dersom den ikke er akseptabel?
    - Hva er det verst tenkelige scenarie for deres selskap?

    Risikovurdering
    - Gjennomfører dere risikovurderinger?
    - Hvordan gjennomføres risikovurderinger?
    - Hvem er ansvarlig for å gjennomføre disse vurderingene?
    - Hvem er involvert i arbeidet med risikovurderinger?
    - Hvilke type risikokategorier vurderer dere?
    - Hva er de mest alvorlige konsekvensene i deres risikovurderinger?

- Avtaler, lover og reguleringer
    - Er dere dataansvarlig eller databehandler i avtale med deres kunder?
    - Er dere dataansvarlig eller databehandler i avtale med deres leverandører?
    - Følger dere noen spesifikke standarder relatert til sikkerhet?
    - Hvordan forholder dere dere til Norm for informasjonssikkerhet?

- Avslutning/ oppsummering

- Er det noe vi burde snakket om som vi ikke har snakket om?

Intervju guide (offentlig)

Dato:	
Navn:	
Rolle:	

Intervjuguide

- Introduksjon
  - Kan du fortelle litt om deg selv og din rolle?
  - Hvor lenge har du jobbet innenfor området velferdsteknologi og digital hjemmeoppfølging?

- Teknologi
  - Kan du fortelle meg litt om den teknologien du har kjennskap tiL?
  - Hvilken erfaring har du med teknologien dere benytter?
  - Hvor mange ulike type teknologier benyttes på pasientene?
  - Hvordan kobles utstyret opp? nettverksoppkobling
    Integrerer sensorer i løsningene – sammenkobling av ulike typer teknologi?

Sensor statistikk

- Cybersikkerhet/ sikkerhet
  - Hva er ditt forhold til cybersikkerhet?
  - Hvordan jobber dere med sikkerhet?
  - Opplever du at sikkerhet er noe dere tar på alvor?
  - Hvem er ansvarlig for sikkerhet i deres løsning/tjeneste? Hvem har ansvar for sikkerhet i løsningene når dere tar dem i bruk?

  - Opplever dere at deres løsning/tjenester er eller kan bli utsatt for uønskede hendelser knyttet til cybersikkerhet?
    - Er det realistisk at løsningene vil bli angrepet?

  - Hvor alvorlig opplever dere at cybersikkerhetshendelser mot deres løsning kan bli?
    - Hvilke konsekvenser kan dette medføre?

  - Har skalering av deres løsninger effekt på cybersikkerhet?
    - Hvis ja, hvordan har skalering effekt på cybersikkerhet?

  - I hvilken grad opplever dere er i stand til å motstå cybersikkerhetshendelser rettet mot deres løsning/tjeneste?

  - Har dere implementert sikkerhetstiltak som dere pålegges?
    - F.eks. relatert til tilgang, passord, standard kryptering, nettverkssementering.

  - Innenfor sikkerhetsarbeidet snakker man i enkelte rammeverk om ulike faser for sikkerhetsarbeidet
    - **Evne til å kjenne helheten i løsningene**, alle avhengigheter, verdikjedene i løsningen fra sensorer til varsling, og sårbarheter knyttet til disse?
    - **Evne til å beskytte**, sørge for at de ulike dele av løsningen er oppdatert, konfigurert på en sikker måte

        - Har dere sikkerhetsverktøy, personell med riktig kompetanse som dekker verdikjeden i løsningen (oppdage nedetid, at deler av løsningen ikke fungerer, oppdage og lukke sårbarheter, avdekke uønsket aktivitet)?
    - **Evne til å oppdage** om sikkerhetshendelser skulle inntreffe i ulike deler av løsningen? Også ute i hjemmet, eks om noen "utforsker" løsningen, angriper sentrale komponenter?
    - **Evne til å respondere** og håndtere om en sikkerhetshendelse skulle inntreffe
  - Opplever dere at det finnes gode ressurser, rammeverk, lover/reguleringer på sikkerhetsområde som styrker cybersikkerhet og eventuelt reduserer risiko innenfor området?
- Risiko
  - **Kan du fortelle meg litt om din forståelse for risiko relatert til cybersikkerhet?**
  - Hvilke trusler er relevante for deres løsning/tjenester/utstyr?
  - Hvilke sårbarheter kan dere bli eksponert for?
    - Hvordan håndterer dere sårbarheter som blir identifisert?
  - Hvilke data lagres i deres løsning?
    - Hvilke type helse- og personopplysninger?
    - Hvilke tilganger har dere til systemene?
  - Hvordan opplever dere risikoen knyttet til deres løsning/tjeneste?
  - Hvordan arbeider dere for å redusere denne risikoen dersom den ikke er akseptabel?
  - Hva er det verst tenkelige scenarie for deres selskap?

  Risikovurdering
  - *Gjennomfører dere risikovurderinger?*
  - *Hvordan gjennomføres risikovurderinger?*
  - *Hvem er ansvarlig for å gjennomføre disse vurderingene?*
  - *Hvem er involvert i arbeidet med risikovurderinger?*
  - *Hvilke type risikokategorier vurderer dere?*
  - *Hva er de mest alvorlige konsekvensene i deres risikovurderinger?*

- Samhandling og samarbeid
  - Hvem er deres sentrale samhandling og samarbeidspartner i arbeidet med velferdsteknologi og digital hjemmeoppfølging?
  - Hvem er involvert i prosessen med å ta i bruk velferdsteknologi og løsninger for digital hjemmeoppfølging?
  - Er det dere som utvikler, kontrollerer, drifter og avlsutter tjenestene?
  - Har dere delt opp deres infrastruktur og nettverk mellom brukere?
  - Har dere gjennomført sikkerhetsrevisjoner eller sikkerhetsgjennomganger av løsninger?

- Underleverandører

  - Hvordan samarbeider dere med leverandører om sikkerhet?
    - På hvilke områder jobber dere sammen?
  - Hvilke forventninge dere innenfor sikkerhet?
    - Til deres leverandører og underleveranødrer?
    - Benytter dere underleverandører for å levere deres løsninger? Hvis JA
    - *Hva leverer deres underleverandører i løsningene?*
    - *Hvordan er ansvaret for sikkerhet distribuert?*
    - *Hvordan er databehandler/ansvarlig fordelt?*
    - *Hvordan samhandler dere?*

- Avtaler, lover og reguleringer
  - Er dere dataansvarlig eller databehandler i avtaleverket?

    - Er dere dataansvarlig eller databehandler i avtale med deres leverandører?
    - Følger dere noen spesifikke standarder relatert til sikkerhet?
    - Hvordan forholder dere dere til Norm for informasjonssikkerhet?

- Avslutning/ oppsummering
    - Hvilke sikkerhetsutfordringer opplever du er størst?
    - Hvilke avveiinger gjøres mellom sikkerhet og kostnad?
    - Er det noe vi ikke har snakket om som vi burde snakke om?
    - Er det noe mer du ønsker å legge til?

Intervju guide (helsetjenesten)

Dato:	
Navn:	
Rolle:	

<u>Intervjuguide</u>

- Introduksjon
    - Kan du fortelle litt om deg selv og din rolle i selskapet?
    - Hvor lenge har du jobbet innenfor området velferdsteknologi og digital hjemmeoppfølging?

- Teknologi
    - Hvilke løsninger for velferdsteknologi og digital hjemmeoppfølging bruker dere i dag?
    - Kan du fortelle meg litt om den teknologien dere benytter?
    - Hvilken erfaring har du med teknologien dere benytter?
    - Hvor mange ulike type teknologier benyttes på pasientene?
    - Hvordan kobles utstyret opp? nettverksoppkobling
Integrerer sensorer i løsningene – sammenkobling av ulike typer teknologi?

- Samhandling og samarbeid
    - Hvem er deres sentrale samhandling og samarbeidspartner i arbeidet med velferdsteknologi og digital hjemmeoppfølging?
    - Hvem er involvert i prosessen med å ta i bruk velferdsteknologi og løsninger for digital hjemmeoppfølging?
    - Er det dere som utvikler, kontrollerer, drifter og avslutter tjenestene?
    - Har dere delt opp deres infrastruktur og nettverk mellom brukere?
    - Har dere gjennomført sikkerhetsrevisjoner eller sikkerhetsgjennomganger av løsninger?

- Cybersikkerhet/ sikkerhet
    - Hva er ditt forhold til cybersikkerhet?
    - Hvordan jobber dere med sikkerhet?
    - Opplever du at sikkerhet er noe dere tar på alvor?
    - Hvem er ansvarlig for sikkerhet i deres løsning/tjeneste? Hvem har ansvar for sikkerhet i løsningene når dere tar dem i bruk?

    - Opplever dere at deres løsning/tjenester er eller kan bli utsatt for uønskede hendelser knyttet til cybersikkerhet?
        - Er det realistisk at løsningene vil bli angrepet?

    - Hvor alvorlig opplever dere at cybersikkerhetshendelser mot deres løsning kan bli?
        - Hvilke konsekvenser kan dette medføre?

    - Har skalering av deres løsninger effekt på cybersikkerhet?
        - Hvis ja, hvordan har skalering effekt på cybersikkerhet?

    - I hvilken grad opplever dere er i stand til å motstå cybersikkerhetshendelser rettet mot deres løsning/tjeneste?

    - Har dere implementert sikkerhetstiltak som dere pålegges?

        - F.eks. relatert til tilgang, passord, standard kryptering, nettverkssementering.

    - Innenfor sikkerhetsarbeidet snakker man i enkelte rammeverk om ulike faser for sikkerhetsarbeidet
        - **Evne til å kjenne helheten i løsningene**, alle avhengigheter, verdikjedene i løsningen fra sensorer til varsling, og sårbarheter knyttet til disse?
        - **Evne til å beskytte**, sørge for at de ulike dele av løsningen er oppdatert, konfigurert på en sikker måte
            - Har dere sikkerhetsverktøy, personell med riktig kompetanse som dekker verdikjeden i løsningen (oppdage nedetid, at deler av løsningen ikke fungerer, oppdage og lukke sårbarheter, avdekke uønsket aktivitet)?
        - **Evne til å oppdage** om sikkerhetshendelser skulle inntreffe i ulike deler av løsningen? Også ute i hjemmet, eks om noen "utforsker" løsningen, angriper sentrale komponenter?
        - **Evne til å respondere** og håndtere om en sikkerhetshendelse skulle inntreffe
    - Opplever dere at det finnes gode ressurser, rammeverk, lover/reguleringer på sikkerhetsområde som styrker cybersikkerhet og eventuelt reduserer risiko innenfor området?

- Risiko
    - **Kan du fortelle meg litt om din forståelse for risiko relatert til cybersikkerhet?**
    - Hvilke trusler er relevante for deres løsning/tjenester/utstyr?
    - Hvilke sårbarheter kan dere bli eksponert for?
        - Hvordan håndterer dere sårbarheter som blir identifisert?
    - Hvilke data lagres i deres løsning?
        - Hvilke type helse- og personopplysninger?
        - Hvilke tilganger har dere til systemene?
    - Hvordan opplever dere risikoen knyttet til deres løsning/tjeneste?
    - Hvordan arbeider dere for å redusere denne risikoen dersom den ikke er akseptabel?
    - Hva er det verst tenkelige scenariet for deres?

    Risikovurdering
    - Gjennomfører dere risikovurderinger?
    - Hvordan gjennomføres risikovurderinger?
    - Hvem er ansvarlig for å gjennomføre disse vurderingene?
    - Hvem er involvert i arbeidet med risikovurderinger?
    - Hvilke type risikokategorier vurderer dere?
    - Hva er de mest alvorlige konsekvensene i deres risikovurderinger?

- Underleverandører
    - Hvordan samarbeider dere med leverandører om sikkerhet?
        - På hvilke områder jobber dere sammen?
    - Hvilke forventninge dere innenfor sikkerhet?
        - Til deres leverandører og underleveranødrer?
        - Benytter dere underleverandører for å levere deres løsninger? Hvis JA
        - *Hva leverer deres underleverandører i løsningene?*
        - *Hvordan er ansvaret for sikkerhet distribuert?*
        - *Hvordan er databehandler/ansvarlig fordelt?*
        - *Hvordan samhandler dere?*

- Avtaler, lover og reguleringer
    - Er dere dataansvarlig eller databehandler i avtaleverket?
    - Er dere dataansvarlig eller databehandler i avtale med deres leverandører?
    - Følger dere noen spesifikke standarder relatert til sikkerhet?

    - Hvordan forholder dere dere til Norm for informasjonssikkerhet?

- Avslutning/ oppsummering
    - Hvilke sikkerhetsutfordringer opplever du er størst?
    - Hvilke avveiinger gjøres mellom sikkerhet og kostnad?
    - Er det noe vi ikke har snakket om som vi burde snakke om?
    - Er det noe mer du ønsker å legge til?

Intervju-guide (expert)

Dato:	
Navn:	
Rolle:	

Intervjuguide

- Introduksjon
  - Kan du fortelle litt om deg selv og din bakgrunn?
  - Hvor lenge har du jobbet innenfor området helse?
  - Hvor lenge har du jobbet innenfor området velferdsteknologi, remote care/ AAL/ IoMT?

- Cybersikkerhet/ sikkerhet
  - Hva er ditt forhold til cybersikkerhet?
  - Hvilken type erfaring har du med cybersikkerhet?
  - Er cybersikkerhet noe du tar på alvor?
  - Hvordan vil du definere en cybersikkerhetshendelse?
  - Hvordan vil du definere en cybersikkerhets risiko?

Cybersikkerhet knyttet til velferdsteknologi, IoMT og remote care:

Risiko relatert til vtek og DHO:

- Kan du fortelle meg litt om din forståelse for cybersikkkerhets risiko relatert til Vtek og DHO?
  - Hvilke trusler er relevante for deres slike løsninger?
  - Hvilke sårbarheter kan de bli eksponert for?
    - Hvordan kan sårbarheter som blir identifisert håndteres?
  - Hvilke data lagres i slike løsninger?
  - Hvordan opplever dere risikoen knyttet til deres løsning/tjeneste?
  - Hvordan kan man jobbe for å redusere denne risikoen?
  - Hva er det verst tenkelige scenarie for denne type teknologi?

- I Norge er de mest vanlige teknologiske løsningene relatert til velderdsteknologi og DHO medisin dispenser, digital trygghetsalarm, elektronisk lås, GPS, videokonferanse, selvrapporteringsskjema, insulinmåling. De følgende spørsmål vil adressere disse teknologiene
  - Hvilke sikkerhetshendelser kan disse utsettes for?
  - Hvilke data?
  - Hvilke trusler?
  - Hvilke sårbarheter er de eksponert for?
  - Hvem er ansvarlig?
  - Er du kjent med noen hendelser som har påvirket teknologien?
  - Hvilke muligheter er det for å utnytte?
  - Hvilke konsekvenser kan dette medføre?
  - Hva er fordelene ved å ta i bruk slik teknologi?
  - Hva er utfordringene?
  - Hvem er ansvarlig for å ivareta sikkerheten i utstyret?
  - Hvordan kan man jobbe for å redusere denne risikoen?

- Hva er det verst tenkelige scenarie for denne type teknologi?

- Cybersikkerhet i helsesektoren
  - Opplever du at helsesektoren tar sikkerhet på alvor?
  - Hvordan opplever du ansvarsdelingen for sikkerhet i helsesektoren?
  - Er du kjent med at helsesektoren er blitt utsatt for uønskede hendelser knyttet til cybersikkerhet?
    - Tenker du at det er realistisk at helsesektoren vil bli utsatt for cybersikkerhetshendelser?
  - Hvor alvorlig opplever du at en cybersikkerhetshendelse kan bli?
  - Hvilke konsekvenser kan forårsakes av cybersikkerhetshendelser?
  - Hvilke verdier/data er det helsesektoren må beskytte?
  - Hva er de største cybersikkerhets truslene for helsesektoren?
  - Hva er de største sårbarhetene for helsesektoren?

  - Innenfor sikkerhetsarbeidet snakker man i enkelte rammeverk om ulike faser for sikkerhetsarbeidet, hvordan vurderer du helsesektoren ut ifra det følgende
    - **Evne til å kjenne helheten i løsningene**, alle avhengigheter, verdikjedene i løsningen fra sensorer til varsling, og sårbarheter knyttet til disse?
    - **Evne til å beskytte**, sørge for at de ulike dele av løsningen er oppdatert, konfigurert på en sikker måte
      - Har dere sikkerhetsverktøy, personell med riktig kompetanse som dekker verdikjeden i løsningen (oppdage nedetid, at deler av løsningen ikke fungerer, oppdage og lukke sårbarheter, avdekke uønsket aktivitet)?
    - **Evne til å oppdage** om sikkerhetshendelser skulle inntreffe i ulike deler av løsningen? Også ute i hjemmet, eks om noen "utforsker" løsningen, angriper sentrale komponenter?
    - **Evne til å respondere** og håndtere om en sikkerhetshendelse skulle inntreffe

Avtaler, lover og reguleringer
  - Hvordan opplever du skillet mellom dataansvarlig, databehandler og dataprosessor?
  - Opplever du at lovverk er tydelig og lett å forstå for de ulike partene i økosystemet?
  - Opplever dere at det finnes gode ressurser, rammeverk, lover/reguleringer på sikkerhetsområde som styrker cybersikkerhet og eventuelt reduserer risiko innenfor området?
  - Er du kjent med norm for informasjonssikkerhet
    - Hvis ja, vennligst utdyp din faglige mening om normen og dens effekt

- Avslutning/ oppsummering
  - Hvilke sikkerhetsutfordringer opplever du er størst?
  - Hvilke avveiinger gjøres mellom sikkerhet og kostnad?
  - Er det noe vi ikke har snakket om som vi burde snakke om?
  - Er det noe mer du ønsker å legge til?

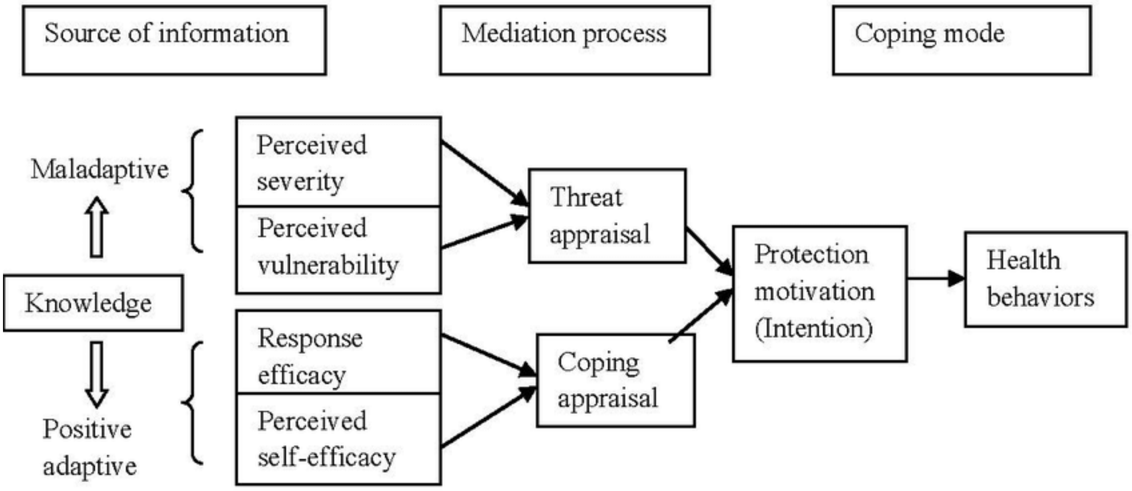

Protection motivation factors:

- Opplever dere at velferdsteknologi og DHO er eller kan bli utsatt for uønsket hendelser knyttet til cybersikkerhet og digitale angrep?
  - (eksponert for risiko ifht. hvilken grad tenker dere velferdsteknologi og DHO er utsatt for trusler ift. cybersikkerhet og digitale angrep)
  - er det realistisk at løsningene vil bli angrepet,
  - finnes det aktører som kan tenkes å angripe systemene? Hvem? Motivasjon, evne?

- Hvor alvorlig opplever dere at angrep og cybersikkerhetshendelser mot velferdsteknologi og DHO kan bli?
  - Hva er konsekvensene for helsehjelpen? – konkretisere
  - Den enkelte innbygger?
  - Utvikling over tid – tjenestetrappen – hva vil dette ha å si for hvor alvorlig angrep kan bli? Hva med om 5 år?

- Opplever kommunen at det finnes gode ressurser, rammeverk, tekniske verktøy på sikkerhetsområdet som kan bidra til å redusere risikoen for cyberangrep? Hvilke verktøy kan dere bygge sikkerheten på?
  - Kjenner dere til Normen eller andre rammeverk for sikkerhet?
  - Hvor godt ligger Normen, ISO27001, CSF "under huden"?
  - Oppleves disse relevant for velferdsteknologi og DHO?
  - Ble normen adrsssert i anskaffelesprosessen?

- I hvilken grad opplever man at sektoren selv er rustet til å motstå cybersikkerhetshendelser rettet mot velferdsteknologi og DHO?
  - Er det forskjeller mellom kommune og RHF sine evner?
-

Innenfor sikkerhetsarbeidet snakker man i enkelte rammeverk om ulike faser for sikkerhetsarbeidet - Identifisere, beskytte, detektere, respondere, gjenopprette

- Evne til å kjenne helheten i løsningene, alle avhengigheter, verdikjedene i løsningen fra sensorer til varsling, og sårbarheter knyttet til disse?
- Evne til å beskytte, sørge for at de ulike dele av løsningen er oppdatert, konfigurert på en sikker måte
- Har sektoren sikkerhetsverktøy, personell med riktig kompetanse som dekker verdikjeden i løsningen (oppdage nedetid, at deler av løsningen ikke fungerer, oppdage og lukke sårbarheter, avdekke uønsket aktivitet)?
- Evne til å oppdage om sikkerhetshendelser skulle inntreffe i ulike deler av løsningen? Også ute i hjemmet, eks om noen "utforsker" gatewayen, angriper sentrale komponenter?
- Evne til å respondere og håndtere om en sikkerhetshendelse skulle inntreffe

- Hvordan oppleves samarbeidet med andre sentrale aktører i arbeidet med sikkerheten i velferdsteknologi og DHO? (utenfor kommunen)
  - Hvem er de/den mest sentrale aktøren?
  - Hvilken rolle opplever du at disse inntar f.eks:
    - Sentrale myndigheter
    - Norsk helsenett
    - KS
    - KommuneCERT
  - På hvilke områder jobber dere sammen?
  - Er det andre aktører relevante for arbeidet med sikkerhet eller som støtter kommunen innenfor sikkerhetsområdet?
  - Er det områder dere opplever at kommunen mangler støtte for å kunne ivareta sikkerheten?
